# Speech-Driven Facial Expression Synthesis Using Emotion-Aware Parametric Facial Descriptors

**DOI:** 10.3390/s26216809

**Published:** 2026-10-28

**Authors:** Sungjung Kim, Jaechern Yoo

**Affiliations:** Department of Electrical and Computer Engineering, Sungkyunkwan University, Suwon 16419, Republic of Korea; kimyjss@skku.edu

**Keywords:** speech emotion recognition, lip descriptor, envelope, facial expression, generative AI

## Abstract

Speech-driven facial expression synthesis aims to generate realistic and emotionally consistent facial motion from speech signals for natural human–computer interaction and expressive virtual agents. This paper presents an emotion-aware framework based on parametric facial descriptors that explicitly models the relationship among speech, emotion, and facial motion. The framework consists of three components. First, a Speech Emotion Recognition (SER) module predicts frame-wise emotion probabilities over six categories: neutral, happy, anger, surprise, sad, and fear. Second, an emotion-conditioned facial descriptor represents interpretable facial expression parameters through a lip descriptor and a head–eye descriptor. The lip descriptor models speech-synchronous mouth articulation using a lookup table of canonical lip geometries, while the head–eye descriptor captures emotion-dependent head pose, eyebrow motion, eyelid activity, and gaze direction. Third, an envelope-driven temporal modulation mechanism generates Animated Facial Expression Parameters (AFP) according to the temporal energy structure of speech. The resulting AFP sequence drives a LivePortrait-based portrait synthesis model to generate emotionally expressive facial images. Experimental results demonstrate visually coherent and temporally consistent facial motion aligned with both speech articulation and emotional state.

## 1. Introduction

Speech-driven facial expression synthesis aims to generate realistic and temporally synchronized facial motion directly from speech signals, and has been widely studied in the computer graphics and vision communities. Early approaches primarily focused on audio-driven visual speech generation, where speech signals are mapped to lip movements or facial animations [1,2]. These methods relied on explicit phoneme-to-viseme correspondence, which limits their ability to capture complex facial dynamics beyond lip articulation. To overcome these limitations, subsequent works introduced trainable and data-driven models for speech animation [3], enabling more flexible and adaptive facial motion generation.

Building upon these developments, recent advances in deep learning have led to significant progress in generating highly realistic talking-head animations. These approaches are commonly referred to as live portrait generation, where a static facial image is animated using driving signals such as speech, video, or motion priors [4,5]. Within this paradigm, end-to-end frameworks have been developed to directly synthesize facial motion from speech signals [6], eliminating the need for handcrafted mappings. Furthermore, recent methods incorporate hierarchical and cross-modal learning strategies to improve visual fidelity and temporal consistency. The use of large-scale audio–visual datasets has also facilitated the learning of speech–face correlations in data-driven facial animation systems [4,5,6].

Despite these advances, most existing methods rely heavily on implicit feature representations, which limit interpretability and controllability. In parallel, neural rendering techniques have advanced rapidly, particularly with the introduction of Neural Radiance Fields (NeRF) [7] and their extensions to dynamic human head avatars [8]. These approaches achieve remarkable photorealism by modeling view-dependent appearance and geometry. However, these approaches also depend on implicit neural representations, making it difficult to directly control semantic facial attributes such as expression and gaze.

Beyond the direct mapping between speech and facial motion, speech signals also contain affective information that can be exploited to estimate the emotional state of a speaker. Recent speech emotion recognition studies have investigated robust emotion estimation from speech by modeling acoustic characteristics and addressing variations in emotional expression across speech data [9]. In particular, recent approaches have explored mel-spectrogram representations and temporal relationships to capture informative emotional cues in speech [10]. These developments indicate that speech emotion information can provide an important conditioning signal for generating more expressive and semantically consistent facial behavior.

As a result, several fundamental challenges remain. First, speech signals are inherently one-dimensional temporal data, whereas facial motion lies on a high-dimensional structured manifold, making the mapping highly complex and underconstrained. Second, raw speech signals contain high-frequency oscillations caused by vocal fold vibrations, which are not directly correlated with meaningful facial motion, often leading to unstable or jittery animation. Third, most existing methods do not explicitly disentangle speech-driven articulation from emotion-dependent facial expressions, limiting their ability to produce expressive and semantically consistent facial behavior. Finally, the reliance on implicit representations reduces interpretability and controllability, which are critical for practical animation systems.

To address these challenges, parametric facial models have been widely adopted due to their structured and interpretable representations [11,12,13]. These models enable explicit control over facial components and have been successfully applied in animation and reconstruction tasks. More recently, hybrid approaches that integrate parametric modeling with learning-based techniques have been proposed to capture dynamic facial expressions and temporal variations [14], bridging the gap between model-based and data-driven methods.

Motivated by these observations, we propose an emotion-aware speech-driven facial expression synthesis framework based on parametric facial descriptors. Unlike prior implicit approaches, the proposed method explicitly separates speech-driven lip articulation from emotion-dependent facial dynamics, enabling more controllable, interpretable, and semantically meaningful facial motion.

Furthermore, we introduce an envelope-driven modulation mechanism that leverages the temporal energy structure of speech signals. By using a smoothed speech envelope rather than raw acoustic signals, the proposed method suppresses high-frequency fluctuations while preserving perceptually relevant temporal dynamics, resulting in more stable and temporally coherent facial motion.

The main contributions of this work are as follows:

- An emotion-aware parametric facial descriptor that disentangles speech-driven lip articulation from emotion-dependent facial dynamics.
- An envelope-based temporal modulation mechanism for stable and smooth facial motion generation.
- A speech-driven facial synthesis framework that combines parametric modeling with learning-based components, improving interpretability, controllability, and temporal coherence.

## 2. Related Work

### 2.1. Speech-Driven Talking Head Generation

Speech-driven talking-head animation has been extensively studied to generate realistic and temporally synchronized facial motion from speech signals. Early approaches relied on explicit phoneme-to-viseme mappings [1,2], which are limited in capturing expressive facial dynamics beyond lip articulation.

To address this limitation, data-driven and learning-based approaches were introduced, enabling models to learn mappings from speech features to facial motion [3]. With the advancement of deep learning, end-to-end frameworks have been developed to directly predict facial motion from audio signals [6], significantly improving realism and generalization.

More recent approaches focus on photorealistic talking-head generation using deep generative models and large-scale audio–visual datasets. Notable works such as You Said That? [15] and rhythmic head motion modeling [16] demonstrate the effectiveness of learning speech–face correlations for generating temporally coherent animations. In addition, live portrait generation methods extend these ideas by animating a static portrait image using speech-driven motion signals [4]. Systems such as Live Speech Portraits [5] further achieve real-time performance through multi-stage pipelines combining audio processing, motion prediction, and neural rendering.

Despite their success, these approaches typically rely on implicit latent representations to model facial motion [4,5,6,15,16]. As a result, they provide limited interpretability and lack explicit control over individual facial components. Moreover, speech-driven articulation and emotion-dependent facial dynamics are often entangled within a single representation, making it difficult to achieve semantically meaningful control.

### 2.2. Neural Rendering and Parametric Head Models

In parallel, neural rendering and head avatar modeling have made significant progress. Neural Radiance Fields (NeRF) [7] and their extensions enable photorealistic rendering by modeling view-dependent appearance and geometry. Recent works extend these approaches to dynamic head avatars [8], enabling animation and reconstruction from monocular inputs.

However, these methods rely on implicit neural representations, which often lack direct interpretability and are computationally expensive. As an alternative, parametric face models [11,12,13] provide structured and interpretable representations of facial geometry and expression, enabling explicit control over facial attributes.

Recent approaches combine parametric models with learning-based techniques to capture temporal dynamics and expressive variations [14]. Additionally, Gaussian-based representations such as GPHM [17] offer efficient rendering and high-fidelity reconstruction by modeling geometry using 3D Gaussian primitives.

Despite these advances, most of these methods focus on visual fidelity and geometric modeling rather than speech-driven animation or emotion-aware control.

### 2.3. Limitations of Existing Approaches

Although substantial progress has been made, several key limitations remain. First, many speech-driven approaches rely on implicit representations [4,5,6,16], limiting interpretability and fine-grained control. Second, speech-driven articulation and emotion-dependent facial dynamics are often entangled, resulting in less expressive and semantically inconsistent facial behavior.

Third, neural avatar models primarily focus on visual realism and reconstruction [7,8,14,17], while lacking mechanisms for modeling speech-driven temporal dynamics. Finally, the direct use of raw speech signals introduces high-frequency fluctuations that are not correlated with meaningful facial motion, often leading to unstable or jittery animations.

To address these limitations, this work proposes an emotion-aware speech-driven facial synthesis framework based on explicit parametric facial descriptors and an envelope-driven temporal modulation mechanism, enabling interpretable, controllable, and temporally stable facial animation.

## 3. Materials and Methods

**Figure 1:**
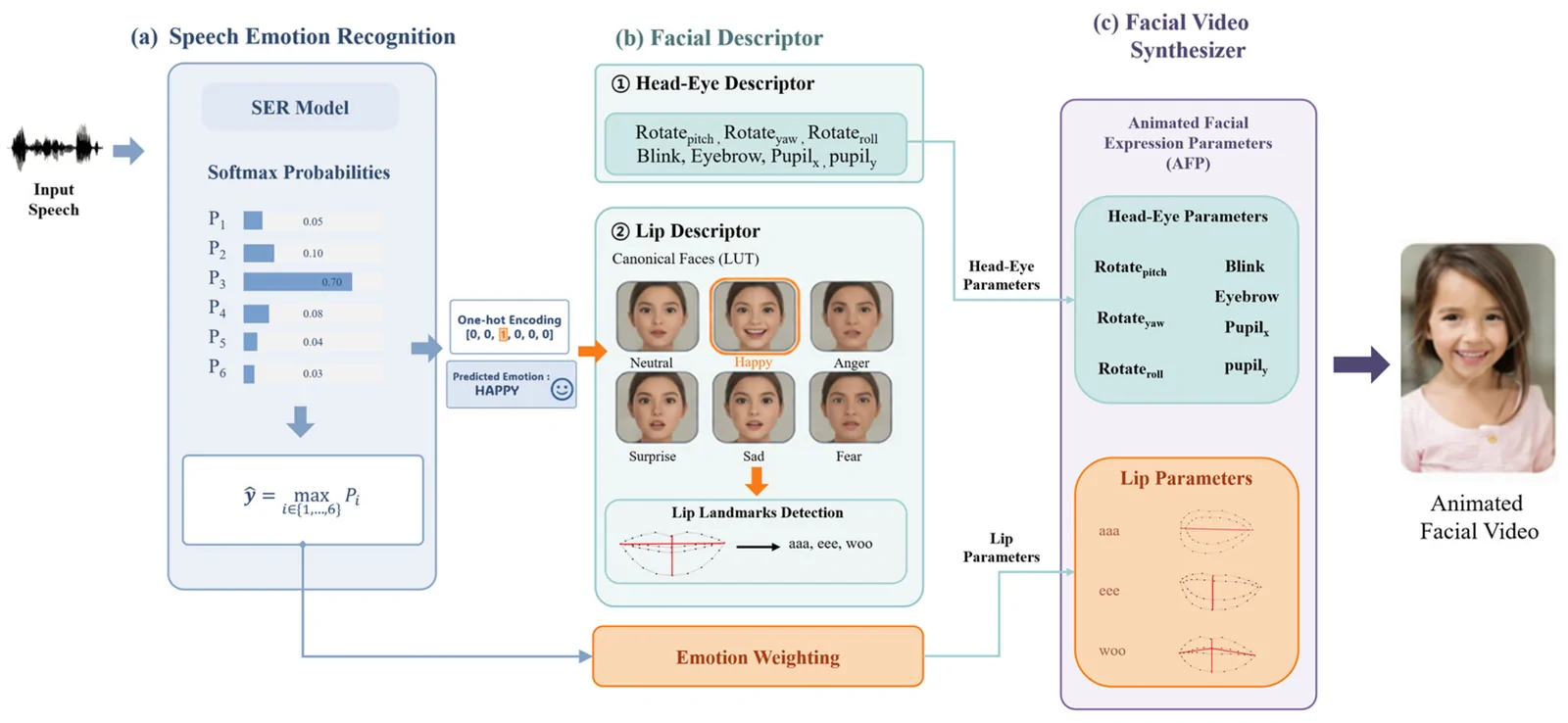
Overview of the proposed speech-driven facial expression synthesis framework. The system consists of three modules: (**a**) Speech Emotion Recognition (SER), which classifies the input speech into six emotional categories and outputs softmax-based emotion probabilities (*P_i_*, where i = 1, 2, … 6); (**b**) an emotion-conditioned facial descriptor composed of ① a head–eye descriptor and ② a lip descriptor that predict facial expression parameters. The lip descriptor utilizes a lookup table (LUT) containing vowel-based lip parameters of emotion-specific canonical faces; and (**c**) a facial video synthesizer that generates a realistic facial animation with Animated Facial expression Parameters (AFP).

This section describes the proposed framework for speech-driven facial expression synthesis. The overall system consists of three main modules: speech emotion recognition (SER), an emotion-conditioned facial descriptor, and a facial video synthesizer (See Figure 1). First, the SER module (Figure 1a) analyzes the input speech signal and classifies it into one of six emotional categories. The recognized emotion is then used to guide the facial descriptor (Figure 1b), which predicts a set of facial expression parameters representing both speech-related articulation and emotion-dependent facial movements. Specifically, the descriptor is decomposed into a lip descriptor, responsible for modeling speech-synchronous lip motion, and a head–eye descriptor, which captures emotion-related head pose, eyebrow motion, eyelid openness, and gaze direction. Finally, the predicted facial expression parameters are used by the facial video synthesizer (Figure 1c) to animate a neutral facial identity and generate a realistic facial video. To ensure smooth temporal evolution of facial expressions, a speech signal envelope–based interpolation mechanism is applied to the predicted parameters, resulting in Animated Facial Expression Parameters (AFP). The detailed implementation of each component is described in the following subsections.

The lookup table (LUT) was constructed using representative emotion-specific facial expressions and vowel articulation images selected through manual visual assessment. The canonical facial expressions were obtained from the CelebA-HQ face image dataset [18] and selected based on their recognizable emotion-related characteristics. The vowel articulation images were manually extracted from the YouTube instructional video entitled Short Vowel Sounds For: a, e, i, o, u (Phonics Lesson) [19], which demonstrates vowel pronunciation through visible mouth movements. Representative video frames were selected at moments corresponding to the pronunciation of individual vowels, based on the visibility and clarity of their characteristic lip configurations. The corresponding lip landmarks were then used to define the lip descriptors stored in the LUT.

### 3.1. Speech Emotion Recognition (SER)

**Figure 2:**
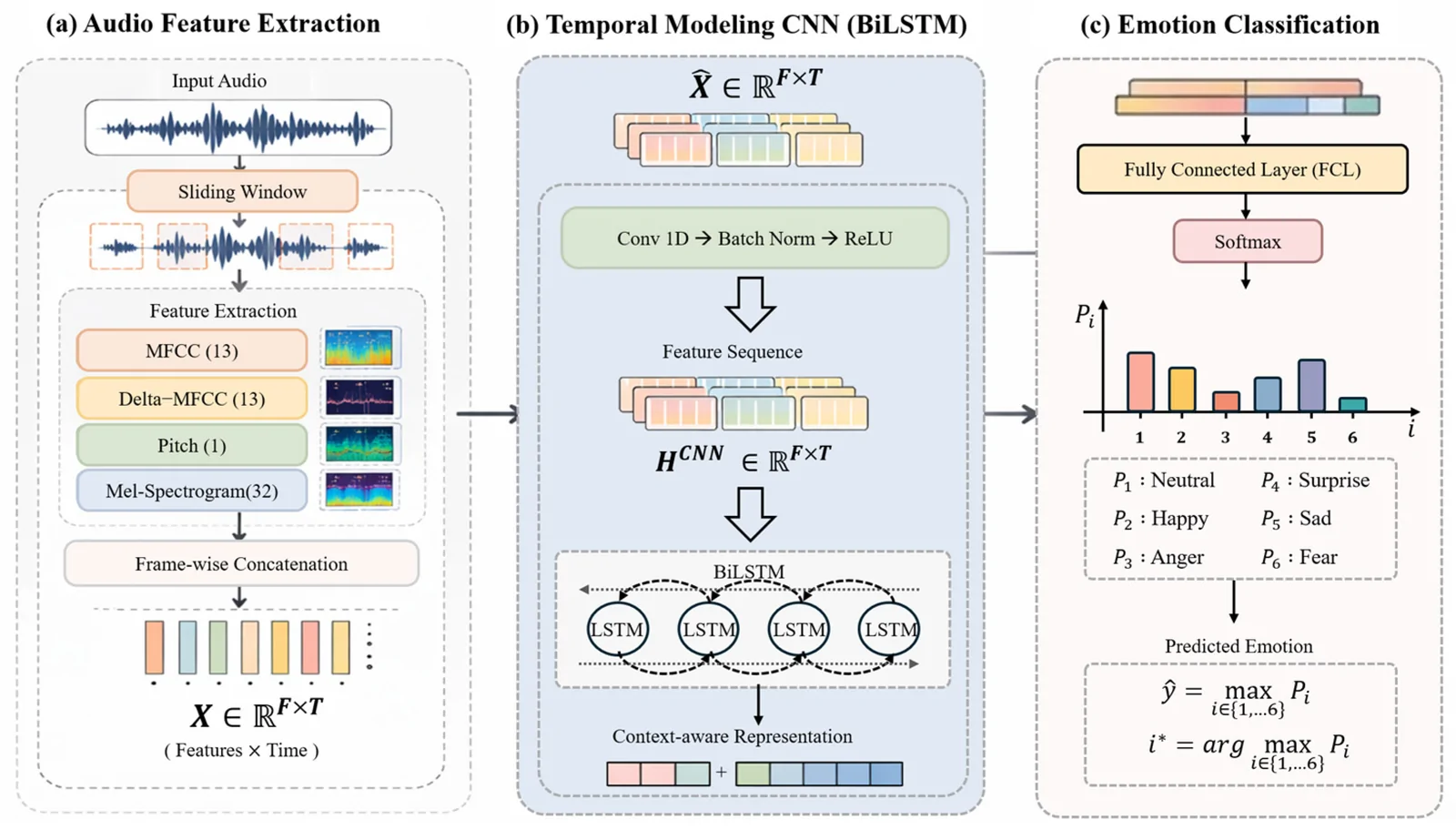
Simplified block diagram of the proposed Speech Emotion Recognition (SER) module. (**a**) Audio feature extraction: the input speech signal is converted into frame-wise acoustic features. (**b**) Temporal modeling: the extracted features are concatenated and fed into a CNN-Bidirectional Long Short-Term Memory (BiLSTM) network to model the temporal dynamics of emotional speech. (**c**) Emotion Classification: the final hidden representation is passed through a fully connected layer (FCL) followed by a softmax classifier to predict one of six emotion categories.

When an input speech signal is provided, the Speech Emotion Recognition (SER) module estimates the underlying emotional state through supervised learning over six predefined emotion classes. As illustrated in Figure 2, the SER pipeline consists of three main stages: (a) acoustic feature extraction, (b) temporal modeling using a Bidirectional Long Short-Term Memory (BiLSTM) network, and (c) emotion classification using a fully connected layer followed by a softmax function.

In the first stage (Figure 2a), several acoustic features are extracted from the input speech signal to capture both spectral and prosodic characteristics of emotional speech. These features include Mel-Frequency Cepstral Coefficients (MFCC), delta-MFCC, pitch, and Mel-spectrogram representations [10,20]. The extracted features are organized as frame-wise feature sequences and serve as inputs to the temporal modeling stage.

The frame-wise acoustic feature sequence is then fed into a Bidirectional Long Short-Term Memory (BiLSTM) network to model temporal dependencies within the speech signal (Figure 2b) [21,22].

The BiLSTM processes the input sequence in both forward and backward directions. The forward LSTM captures contextual information from preceding frames, while the backward LSTM captures information from succeeding frames. By integrating information from both temporal directions, the network produces a context-aware representation that reflects the overall emotional dynamics of the utterance.

The use of BiLSTM is motivated by its effectiveness in modeling sequential data with long-range temporal dependencies. Emotional cues in speech are often distributed across multiple frames rather than confined to isolated segments. The recurrent structure of LSTM units enables the network to retain relevant contextual information over extended time intervals while alleviating the vanishing gradient problem. This capability makes BiLSTM particularly suitable for capturing the prosodic and dynamic characteristics associated with emotional speech.

Finally, the learned representation is passed to a fully connected layer that outputs class probabilities (*P_i_*) over the six emotion categories using a softmax activation function, where the integer i is an index variable indicating one of the six predefined emotion categories, i.e., *i* ∈ {1, 2, …, 6}.

The maximum emotion confidence score $\hat{y}$, together with the predicted emotion index i*, is subsequently used to condition the facial descriptor module described in Section 3.2. They are defined as

(1) $$\hat{y}=\underset{i\in \{1,\ldots ,6\}}{\max}P_{i}\;\mathrm{and}\;i^{*}=\arg\underset{i\in \{1,\ldots ,6\}}{\max}P_{i}$$

where *P_i_* denotes the softmax probability of the *i*-th emotion class.

#### 3.1.1. Feature Extraction

**Figure 3:**
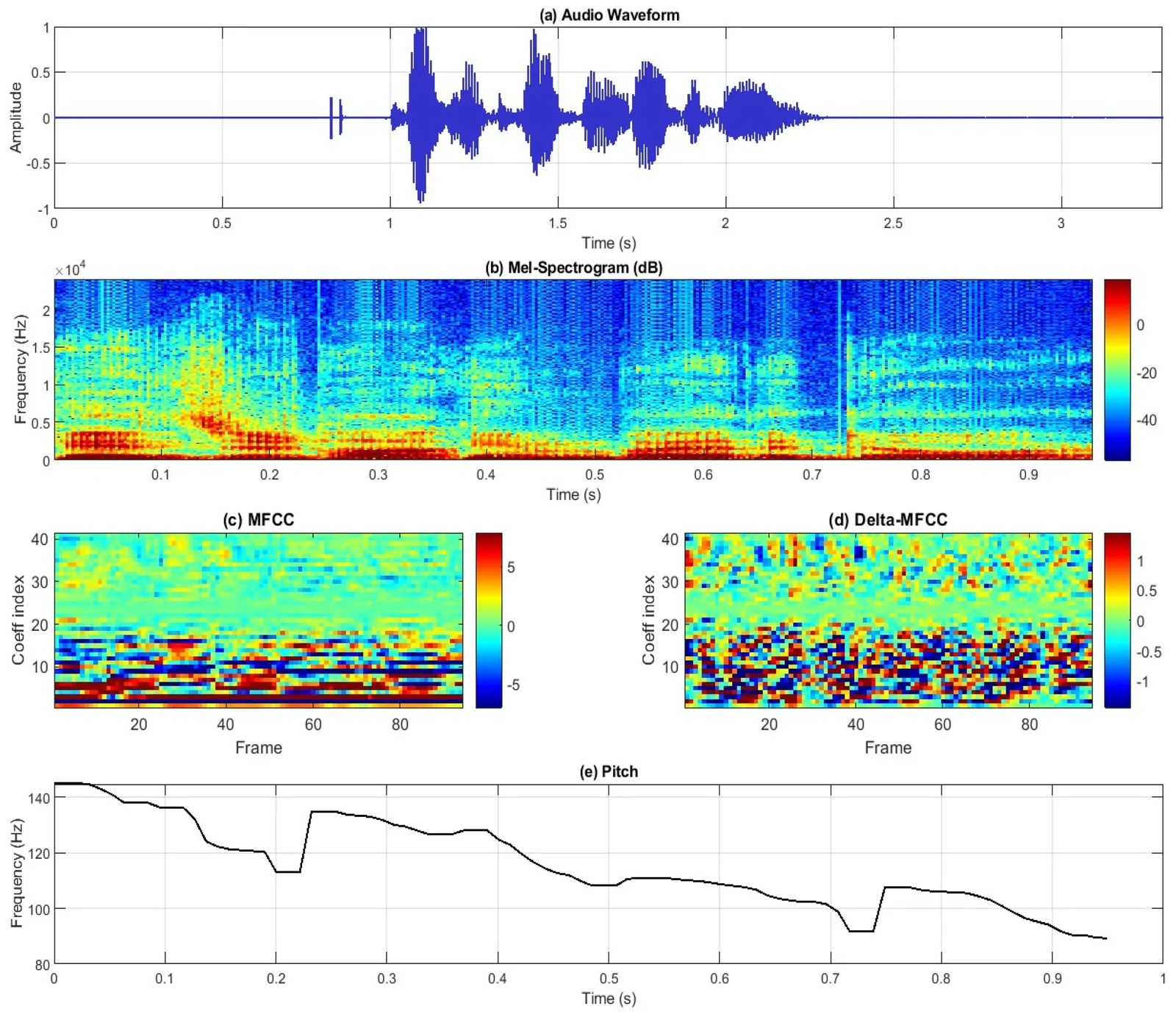
Examples of acoustic feature representations extracted from an input speech signal: (**a**) raw audio waveform, (**b**) Mel-spectrogram, (**c**) MFCC coefficients, (**d**) Delta-MFCC, and (**e**) Pitch contour (fundamental frequency).

Four acoustic feature types are extracted from the input speech signal using a high-resolution audio feature extraction procedure with a 40 ms Hamming window and 20 ms frame overlap (See Figure 3). The extracted features capture both spectral and prosodic characteristics of emotional speech.

The following acoustic features are computed:

(1) 13 Mel-Frequency Cepstral Coefficients (MFCCs), denoted by MFCC*_vec_* = [*c*_1_, *c*_2_, …, *c*_13_], which represent a compact description of the spectral envelope of speech. The MFCC extraction process involves the following steps [20]:

1. Short-Time Fourier Transform (STFT) of each speech frame
2. Application of a Mel filterbank to the power spectrum ${\left|X_{\mathrm{in}}(f)\right|}^{2}$:

(2) $$S_{n}=\sum_{f}{\left|X_{\mathrm{in}}\left(f\right)\right|}^{2}\;H_{n}(f)$$

where *S_n_* = energy of the *n*-th Mel filter.

3. Logarithmic compression of the Mel filterbank energies: log(*S_n_*)
4. Discrete Cosine Transform (DCT) applied to the log Mel energies (log(*S_n_*))

The DCT converts the log Mel spectral energies into cepstral coefficients:

(3) $$c_{k}=\sum_{n=1}^{N}\log\left(S_{n}\right)\cos\left[\frac{\mathrm{\pi k}}{N}(n-0.5)\right]\;k=1,\;\ldots ,\;13$$

where *S_n_* denotes the energy of the *n*-th Mel filter, *N* represents the number of Mel filters (*N* = 32), and *k* denotes the cepstral coefficient index. In this study, only the first 13 coefficients were retained to represent the spectral envelope of each speech frame, as the lower-order cepstral coefficients effectively capture the spectral envelope of speech while suppressing high-frequency spectral variations and noise.

(2) 13 Delta-MFCC Coefficients, denoted by ΔMFCC*vec* = [Δ*c*1, Δ*c*2, …, Δ*c*13], which represent the temporal derivatives of the MFCC features. Delta coefficients capture the rate of change in spectral features across neighboring frames and are computed using finite-difference approximation:

(4) $$\Delta c_{k}\left(t\right)=\frac{\sum_{n=1}^{N}n(c_{k}\left(t+n\right)-c_{k}\left(t-n\right))}{2\sum_{n=1}^{N}n^{2}}$$

where *t* denotes the frame index. By incorporating temporal dynamics of speech, delta-MFCC features provide important cues for modeling emotional prosody and articulation changes over time.

(3) Pitch, which represents the fundamental frequency (*F*0) of the speech signal at frame *t*. Pitch reflects vocal fold vibration rate and plays a critical role in expressing emotional states in speech. For example, emotions such as happiness or surprise often exhibit higher pitch values, while sadness tends to be associated with lower pitch. In this study, the pitch contour was extracted frame-wise and used as a prosodic feature for emotion recognition.

(4) 32 Mel-Spectrogram Features, denoted by **S**(*t*) = [log*S*_1_(*t*), log*S*_2_(*t*), …, log*S_N_*(*t*)], which represent the log-scaled Mel filter bank energies of the speech spectrum. The Mel-spectrogram provides a time–frequency representation that reflects the perceptual frequency resolution of human hearing. Compared with MFCCs, which provide a compact cepstral representation, Mel-spectrogram features preserve more detailed spectral structure and therefore complement MFCC-based representations in capturing emotional characteristics of speech [10].

After feature extraction, global Z-score normalization is applied using the mean and standard deviation computed from the entire training set:

(5) $${\hat{x}}_{f,t}=\frac{x_{f,t}-\mu_{f}}{\sigma_{f}}$$

where *x_f_*_,*t*_ denotes the *fth* acoustic feature at frame *t*, and *μ_f_* and *σ_f_* represent the mean and standard deviation of the *fth* feature computed from the training set.

Finally, each speech utterance is represented as a normalized time–feature matrix $\hat{Χ}\in R^{F\times T}$, where *F* = 59 denotes the total number of acoustic features and *T* represents the number of temporal frames.

The normalized feature sequence is then provided as input to the temporal modeling network described in the following subsection.

#### 3.1.2. Temporal Emotion Modeling Network

The normalized acoustic feature sequence described in the previous subsection serves as the input to the Speech Emotion Recognition (SER) model, which learns temporal patterns of emotional speech. Let the normalized feature matrix be

(6) $$\hat{X}=\frac{X-\mu }{\sigma }$$

where **X** ∈ ℝ*^F^*^×*T*^ denotes the original feature matrix, and ***μ*** and ***σ*** represent the feature-wise mean and standard deviation computed from the training set. The normalized sequence $\hat{X}$ is then processed by a temporal modeling network to estimate the emotional state of the input speech signal.

To jointly model local spectral structures and long-range temporal dependencies, a hybrid CNN–BiLSTM architecture is employed. The convolutional layers capture short-term spectral–temporal patterns from the acoustic feature sequence, while the BiLSTM summarizes global emotional dynamics across the entire utterance.

**Figure 4:**
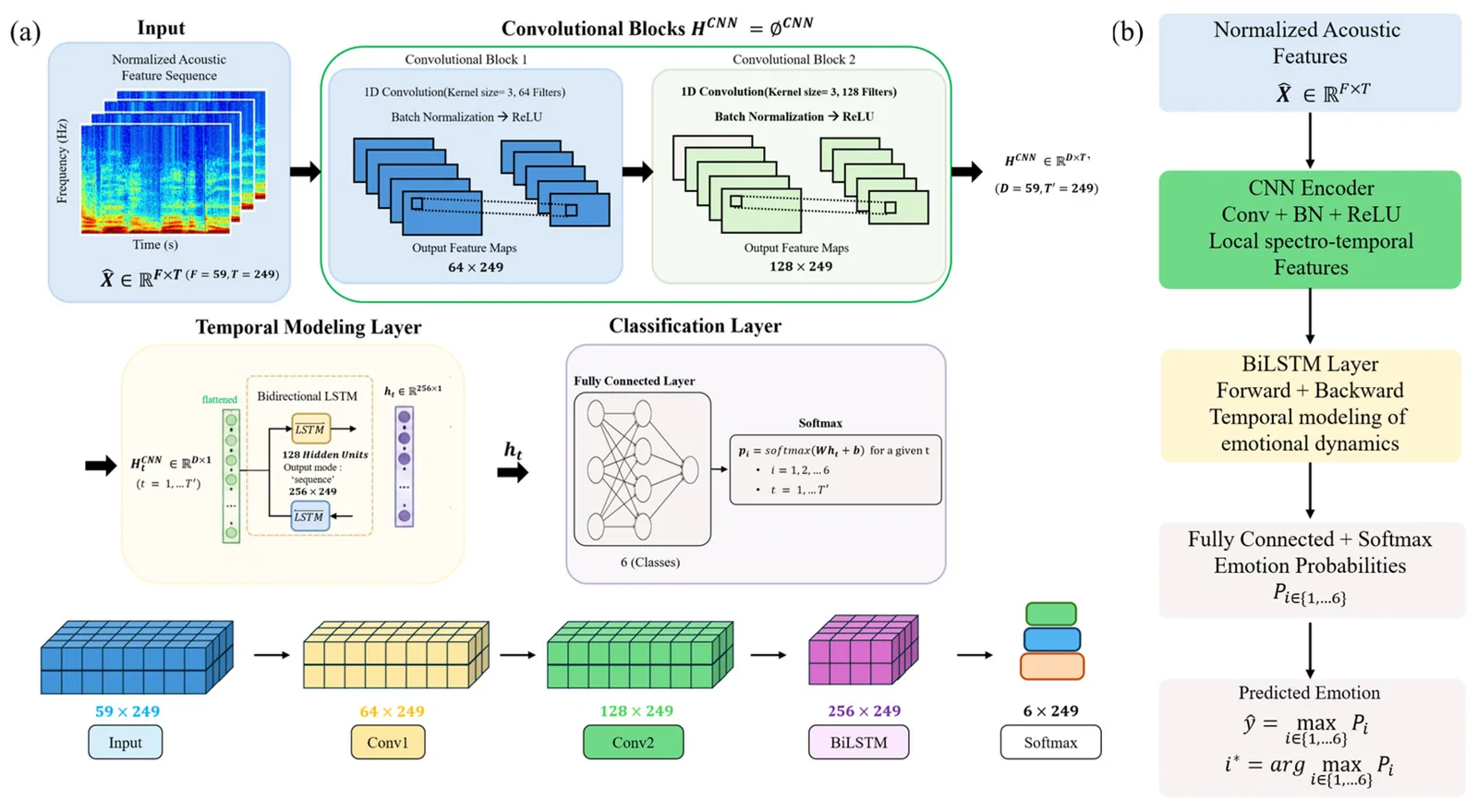
Simplified architecture of the Speech Emotion Recognition (SER) network: (**a**) detailed architecture of the proposed CNN–BiLSTM model, including the convolutional blocks, temporal modeling layer, and classification layer; (**b**) overall processing flow from normalized acoustic features through the CNN encoder, BiLSTM layer, and fully connected softmax classifier to the predicted emotion. The convolutional layers capture local spectro-temporal patterns, while the BiLSTM layer models long-range temporal dependencies across the utterance. Finally, the softmax classifier produces the posterior probability distribution over six emotion classes.

The experimentally optimized SER network through the ablation study consists of the following components (see Figure 4):

The CNN–BiLSTM network was trained using the Adam optimizer with a cross-entropy loss function. The maximum number of training epochs was set to 60, with a mini-batch size of 32 and an initial learning rate of 0.001. To stabilize the training process and mitigate overfitting, a gradient threshold of 1, an L2 regularization coefficient of 0.003, and a dropout rate of 0.1 were employed. The training samples were shuffled at the beginning of every epoch.

The training hyperparameters were empirically determined through preliminary experiments. In particular, the maximum number of training epochs was set to 60, as classification accuracy showed no meaningful improvement beyond this point in preliminary experiments with up to 100 epochs. Other hyperparameters were manually adjusted based on the observed model performance.

(1) Convolutional Block 1 and 2

Given the normalized acoustic feature sequence $\hat{X}\in R^{F\times T}$, the convolutional layers first extract local spectral–temporal patterns:

(7) $$H^{\mathrm{CNN}}=\emptyset_{\mathrm{CNN}}\left(\hat{X}\right)$$

where *ϕ*_CNN_(⋅) denotes the stacked convolution, batch normalization, and ReLU activation layers, and $H^{\mathrm{CNN}}\in R^{D\times T^{\prime }}$ represents the resulting high-level feature sequence. Here, *D* denotes the number of output feature channels produced by the convolutional block, and *T*′ denotes the number of temporal frames after convolutional processing.

(2) Temporal Modeling Layer

The extracted sequence is then processed by a Bidirectional Long Short-Term Memory (BiLSTM) layer to capture temporal dependencies in both forward and backward directions [19,20]:

(8) $$h_{t}=\left[\vec{\mathrm{LSTM}}{(H}_{t}^{\mathrm{CNN}}),\;\overset{\leftarrow }{\mathrm{LSTM}}{(H}_{t}^{\mathrm{CNN}})\right],\;t=1,2,\ldots ,T^{\prime }$$

where *h_t_* denotes the bidirectional hidden representation at time step *t*, and [ · , · ] indicates the concatenation of the forward and backward LSTM hidden states. *H* denotes the hidden dimension of each directional LSTM.

(3) Classification Layer

Since the RAVDESS dataset provides utterance-level emotion annotations, each speech utterance was normalized to a fixed duration of 5 s, and its corresponding emotion label was propagated to all acoustic frames extracted from that utterance. Specifically, for an utterance containing T frames, the same emotion label was assigned to all T frames to construct the frame-level target sequence. The CNN–BiLSTM network was trained using these propagated labels with a cross-entropy loss function and configured in sequence output mode to generate frame-wise emotion probability estimates.

The bidirectional hidden representation ***h****_t_* at each time step is passed through a fully connected layer followed by a softmax classifier to produce frame-wise emotion probabilities, where the BiLSTM’s output is in sequence mode:

*P_i_* = *softmax*(***Wh****_t_* + ***b***) for a given *t* (9)

where ***W*** and ***b*** denote the weight matrix and bias vector of the classification layer, respectively, and *P_i_* represents the predicted emotion probability vector at time step *t*.

Finally, the maximum emotion confidence score at time step *t* is obtained as:

(10) $$\hat{y}=\underset{i\in \{1,\ldots ,6\}}{\max}P_{i}$$

where *P_i_* denotes the predicted probability of the *i*-th emotion class.

Importantly, the frame-wise $\hat{y}$ and its emotion index are subsequently used to condition the facial descriptor module described in Section 3.2. This probabilistic representation enables emotion-aware conditioning and supports smooth emotional transitions in the later stages of the speech-driven facial synthesis pipeline.

### 3.2. Emotion-Conditioned Facial Descriptor

To bridge the Speech Emotion Recognition (SER) module and the facial synthesis framework, we introduce an emotion-conditioned facial descriptor composed of ten interpretable parameters. These parameters encode emotion-related facial variations and provide a compact representation for controlling facial animation.

As summarized in Table 1 and Table 2, each parameter is defined with minimum and maximum allowable ranges, which constrain the physically plausible deformation of the facial model. The proposed descriptor is divided into two complementary groups:

- Lip descriptor, which models emotion-dependent mouth deformations associated with speech articulation and affective expression (Table 1).
- Head–eye descriptor, which represents global head orientation and eye gaze movements, including head rotations, eyelid closure, eyebrow motion, and pupil displacement (Table 2).

Together, these components form a 10-dimensional parametric facial descriptor that enables continuous and controllable emotional facial expression synthesis. The lip descriptor primarily captures speech-synchronous articulatory motion, while the head–eye descriptor conveys emotion-dependent facial dynamics, allowing the generated facial animation to reflect both the linguistic content and the emotional state of the input speech signal. The detailed formulation of the lip and head–eye descriptors is described in the following subsections.

#### 3.2.1. Lip Descriptor

Facial emotion is closely associated with variations in lip shape and mouth configuration. To capture emotion-dependent mouth deformation patterns, we define a set of lip-related parameters that represent key geometric changes in the mouth region.

In this study, three vowel-based lip parameters—aaa, eee, and woo—are adopted to model the principal modes of lip deformation, corresponding to vertical mouth opening, horizontal lip stretching, and lip rounding or forward protrusion, respectively, as summarized in Table 1. Each parameter corresponds to a specific geometric deformation component of the mouth region and plays an important role in modeling expressive facial motion.

To construct a reliable mapping between emotion categories and lip deformation patterns, a set of emotion-specific canonical facial images is prepared. Each canonical facial image corresponds to a predefined emotion class and is associated with a full emotion probability of 1 for its corresponding class. For each canonical image, the corresponding vowel-based lip parameters, denoted by $\theta_{\left(i\right)}^{\mathrm{vowel}}$, are extracted and stored in a lookup table (LUT).

**Figure 5:**
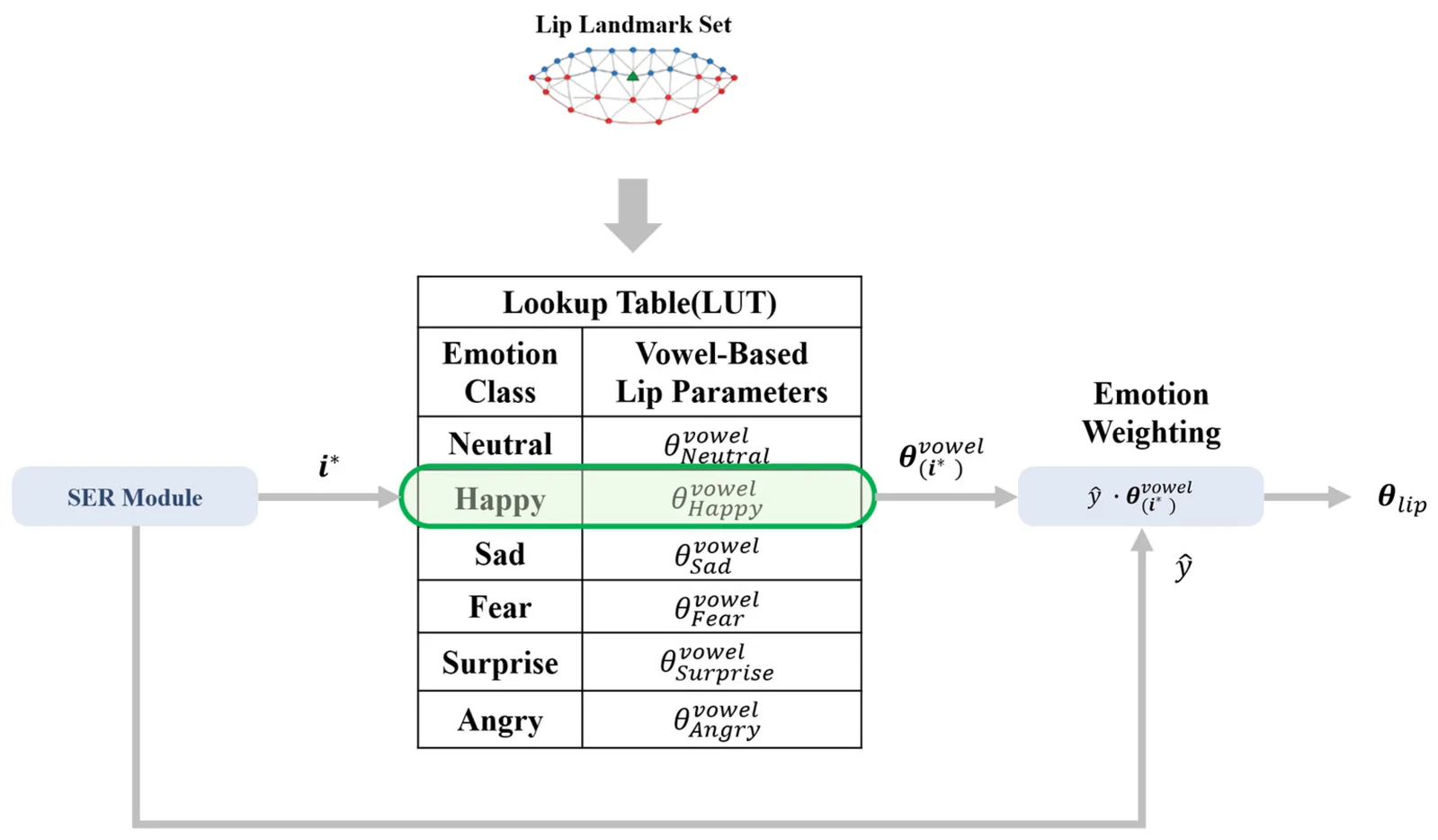
Emotion-conditioned lip parameter selection using a lookup table (LUT). The Speech Emotion Recognition (SER) module predicts the emotion index i*, which is used to access the corresponding vowel-based lip parameter vector $\theta_{\left(i^{*}\right)}^{\mathrm{vowel}}$ stored in the LUT. The retrieved parameter vector is then modulated by the maximum emotion confidence score $\hat{y}$ through an emotion-weighting operation to produce the final lip parameter vector ***θ****_lip_* used for facial animation.

During inference, the LUT is accessed using the one-hot encoded emotion (i^*^) produced by the Speech Emotion Recognition (SER) module. Then the retrieved parameter vector $\theta_{\left(i^{*}\right)}^{\mathrm{vowel}}$ is weighted by the maximum emotion confidence score $\left(\hat{y}\right)$ to reflect the emotional characteristics of the input speech signal (See Figure 5). This mechanism enables the lip descriptor to generate continuous lip deformation patterns that are consistent with the predicted emotional state.

(1) Lip Landmarks

To establish a consistent geometric relationship between the six emotion categories and the vowel-based lip parameters, two groups of reference facial images are prepared. The first group consists of six emotion-specific facial images, each corresponding to one of the predefined emotion classes. The second group consists of three vowel articulation images, captured while pronouncing the vowels aaa, eee, and woo, respectively.

For each reference image, a dense set of facial landmarks is detected to obtain a detailed geometric representation of the facial structure. Each landmark is represented by a two-dimensional coordinate vector indicating its spatial position in the image plane [23].

**Figure 6:**
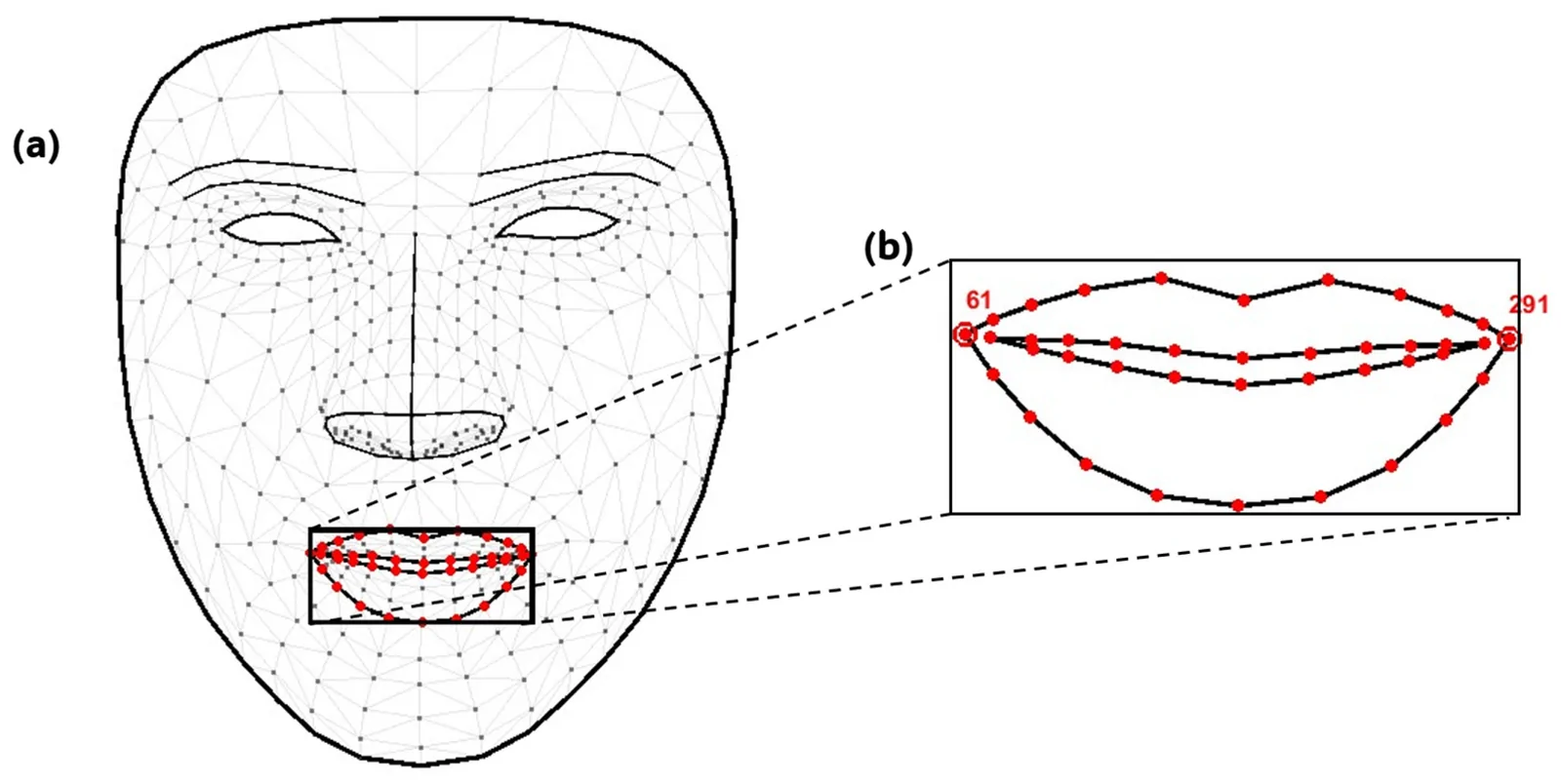
Facial landmark detection and mesh reconstruction using a 468-point facial landmark model [23]. The lip region is extracted as a subset of the facial mesh for geometric analysis. (**a**) Full facial landmark detection. (**b**) Extracted lip landmarks, where landmarks 61 and 291 correspond to the right and left corners of the mouth, respectively.

Among the detected facial landmarks, those corresponding to the mouth region are selected to form a lip landmark set. As illustrated in Figure 6, the lip region is represented as a subset of facial landmarks extracted from the reconstructed facial mesh. Let *L*_lip_ ⊂ *L*_face_ denote the subset of landmarks associated with the lip contour, where *L*_face_ represents the full set of facial landmarks.

The extracted lip landmarks define the geometric structure of the mouth region and enable the estimation of vowel-based lip parameters for emotion-specific facial images by measuring displacements relative to the lip landmarks of vowel articulation images. These landmarks provide a geometric representation of lip shape and configuration, serving as the foundation for parameter estimation.

In particular, variations in lip landmark geometry capture the articulatory patterns associated with the vowels aaa, eee, and woo, corresponding to vertical mouth opening, horizontal lip stretching, and lip rounding, respectively.

The extraction of vowel-based lip parameters from these landmarks is described in the following subsection.

(2) Vowel-based Lip Parameters

To facilitate the estimation of vowel-based lip parameters from emotion-specific facial images, their landmark sets are geometrically aligned with those of the vowel articulation images. In this study, landmark 61 (or 291 for the opposite side) is used as the geometric reference for alignment.

Let ***L****^emotion^*(*x*, *y*) denote the lip landmark set of an emotion-specific facial image, and ***L****^vowel^*(*x*, *y*) denote the lip landmark set of a vowel articulation image, where

*emotion* ∈ {neutral, happy, anger, surprise, sad, fear} *and vowel* ∈ {*aaa*, *eee*, *woo*} (11)

To compensate for global positional differences between the two landmark sets, the emotion-specific landmarks are translated so that landmark **61** coincides with that of the corresponding vowel articulation image. The aligned lip landmark set is defined as

(12) $$L_{\mathrm{align}}^{\mathrm{emotion}}\left(x,y\right)=L^{\mathrm{emotion}}\left(x,y\right)+\left[L^{\mathrm{vowel}}\left(x_{61},y_{61}\right)-L^{\mathrm{emotion}}\left(x_{61},y_{61}\right)\right]$$

After alignment with respect to landmark **61**, the geometric difference between $L_{\mathrm{align}}^{\mathrm{emotion}}\left(x,y\right)$ and ***L****^vowel^*(*x*, *y*) is quantified using the mean Euclidean distance

(13) $$D_{\mathrm{emotion}}^{\mathrm{vowel}}={∥L^{\mathrm{vowel}}(x,y)-L_{\mathrm{align}}^{\mathrm{emotion}}(x,y)∥}_{2}$$

This yields a distance set of vowel articulation images for each emotion-specific facial image:

(14) $$D=\left\{D_{\mathrm{emotion}}^{\mathrm{aaa}},D_{\mathrm{emotion}}^{\mathrm{eee}},D_{\mathrm{emotion}}^{\mathrm{woo}}\right\}$$

To estimate the relative geometric similarity between each emotion-specific lip configuration and the corresponding vowel articulation, the geometric distances are transformed into individual similarity scores bounded between 0 and 1 using Equation (15). A smaller normalized distance produces a higher similarity score. These scores are independently bounded and are not required to sum to unity, as they are subsequently scaled to the allowable ranges of the corresponding vowel-based lip parameters.

(15) $${\mathrm{Score}}_{\mathrm{emotion}}^{\mathrm{vowel}}=1-\frac{D_{\mathrm{emotion}}^{\mathrm{vowel}}}{D_{\mathrm{emotion}}^{\mathrm{aaa}}+D_{\mathrm{emotion}}^{\mathrm{eee}}+D_{\mathrm{emotion}}^{\mathrm{woo}}}$$

The normalized score reflects the relative contribution of each lip parameter to the emotion-specific facial image.

Let ***S****_emotion_* denote the set of normalized vowel-based scores associated with a given emotion-specific facial image:

(16) $$S_{\mathrm{emotion}}=\left\{{\mathrm{Score}}_{\mathrm{emotion}}^{\mathrm{aaa}},{\mathrm{Score}}_{\mathrm{emotion}}^{\mathrm{eee}},{\mathrm{Score}}_{\mathrm{emotion}}^{\mathrm{woo}}\right\}$$

Since each lip parameter must lie within the predefined ranges specified in Table 1, the normalized scores are scaled to obtain the final vowel-based lip parameters:

(17) $$\theta_{\mathrm{emotion}}^{\mathrm{vowel}}={\mathrm{Score}}_{\mathrm{emotion}}^{\mathrm{vowel}}\cdot \max\left(\mathrm{vowel}\right)+\left(1-{\mathrm{Score}}_{\mathrm{emotion}}^{\mathrm{vowel}}\right)\cdot \min\left(\mathrm{vowel}\right)$$

where max(*vowel*) and min(*vowel*) denote the maximum and minimum allowable values of each vowel-based lip parameter, as specified in Table 1. To enable efficient emotion-driven facial expression synthesis, the resulting lip parameters are stored in a lookup table (LUT):

(18) $$\theta_{\left(i\right)}^{\mathrm{vowel}}=\left[\theta_{\mathrm{emotion}}^{\mathrm{aaa}},\theta_{\mathrm{emotion}}^{\mathrm{eee}},\theta_{\mathrm{emotion}}^{\mathrm{woo}}\right]$$

where the integer *i* denotes the corresponding emotion category index. During inference, the LUT is accessed using the predicted emotion index i* obtained from the SER module. Then the corresponding lip parameter vector $\theta_{\left(i^{*}\right)}^{\mathrm{vowel}}$ is retrieved from the LUT and is weighted by the maximum emotion confidence score $\left(\hat{y}\right)$ to reflect the emotional characteristics of the input speech signal (See Figure 5):

(19) $$\theta_{\mathrm{lip}}=\hat{y}\cdot \theta_{\left(i^{*}\right)}^{\mathrm{vowel}}$$

where $\theta_{\mathrm{lip}}=[\theta_{\mathrm{lip}}^{\mathrm{aaa}},\theta_{\mathrm{lip}}^{\mathrm{eee}},\theta_{\mathrm{lip}}^{\mathrm{woo}}]$ is an emotion-weighted lip parameter vector, $\hat{y}=\underset{i\in \{1,\ldots ,6\}}{\max}P_{i}$, and $i^{*}=\arg\underset{i\in \{1,\ldots ,6\}}{\max}P_{i}$.

#### 3.2.2. Head-Eye Descriptor

While lip deformation plays a primary role in conveying emotional expressions, global facial orientation and gaze behavior also provide important cues for producing natural and believable facial animation. To incorporate these effects, we introduce a head–eye descriptor that models parameters such as rigid head motion, eyelid dynamics, eyebrow movement, and pupil displacement. Although these head–eye pose parameters contribute less directly to emotional expression than the lip parameters, they play an important role in enhancing the realism and naturalness of facial animation. Together with the lip parameters described in Section 3.2.1, they form the complete 10-dimensional facial expression parameter set used in this study.

The proposed head–eye descriptor consists of seven parameters: *Rotate_pitch_*, *Rotate_yaw_*, *Rotate_roll_*, *Blink*, *Eyebrow*, *Pupil_x_*, and *Pupil_y_*, as summarized in Table 2. Each parameter corresponds to an interpretable facial motion component that contributes to expressive facial behavior during speech. These facial motion components are consistent with the facial muscle movements and behavioral cues commonly described in the Facial Action Coding System (FACS) [24].

**Figure 7:**
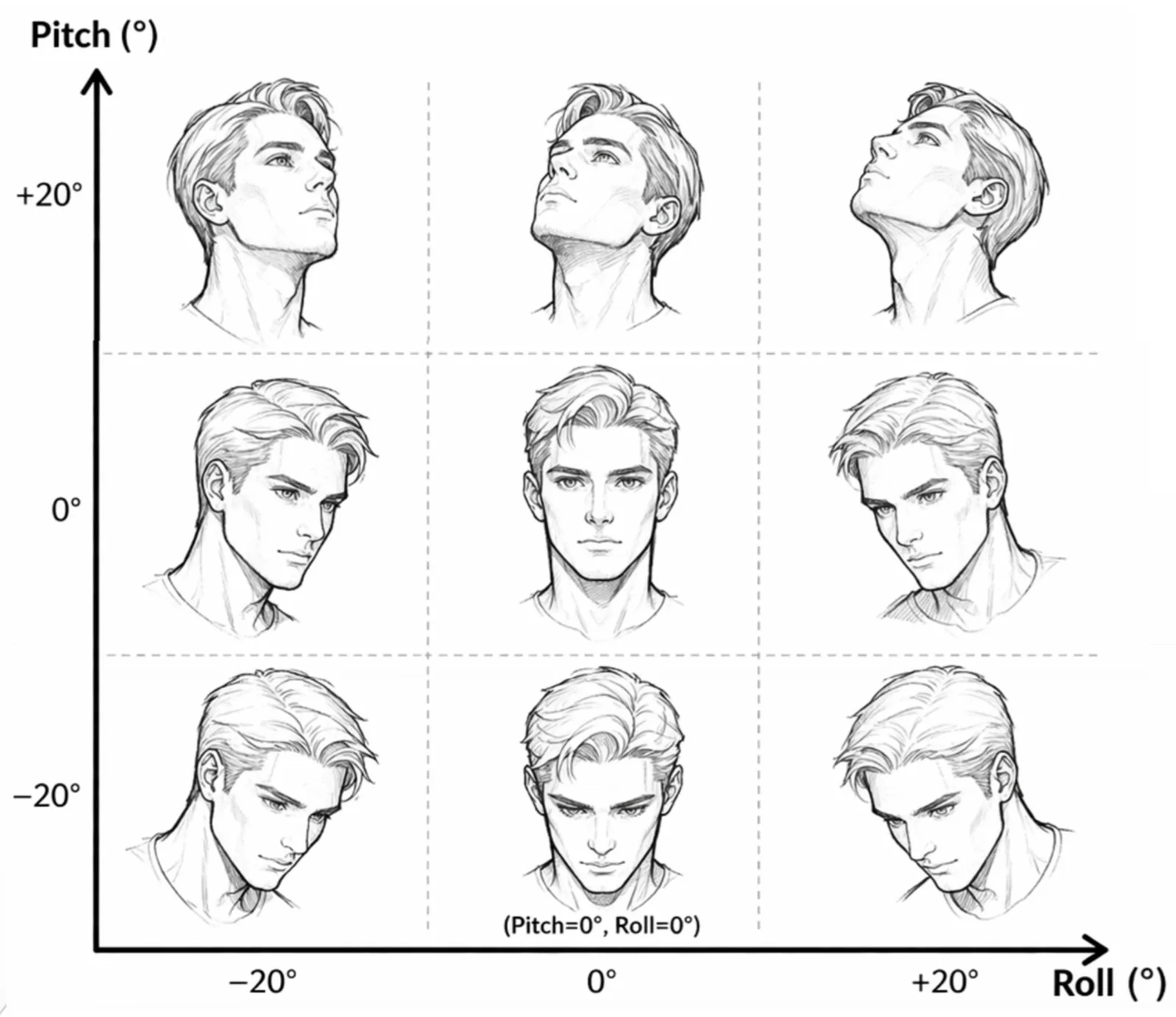
Examples of head pose variations generated using the proposed rotation parameters. The horizontal axis represents roll rotation (−20°, 0°, +20°), while the vertical axis corresponds to pitch rotation (−20°, 0°, +20°). Yaw is fixed at 0° in all examples.

The three rotation parameters model global head orientation. Specifically, *Rotate_pitch_* controls vertical head tilt (up–down motion), *Rotate_yaw_* represents horizontal head turning (left–right movement), and *Rotate_roll_* captures in-plane head rotation. By adjusting these parameters, the system can generate natural head movements that often accompany emotional speech. Examples of head pose variations produced by these parameters are illustrated in Figure 7.

In addition to head motion, eye-related dynamics are modeled through dedicated parameters. The Blink parameter controls eyelid closure intensity, enabling the simulation of blinking behavior. The Eyebrow parameter represents vertical eyebrow displacement and models eyebrow raising or lowering during expressive facial movements. Furthermore, gaze direction is represented using the two parameters *Pupil_x_* and *Pupil_y_*, which correspond to horizontal and vertical pupil displacement, respectively. These parameters allow the system to adjust gaze direction independently of head orientation.

The integration of these head–eye pose parameters with the vowel-based lip parameters enables the generation of coherent and expressive facial motion that reflects both speech articulation and emotional state. The combined formulation of these parameters is described in the following subsection.

#### 3.2.3. LivePortrait Image Synthesis

Together with the lip parameters defined in Section 3.2.1, the head–eye pose parameters described in Section 3.2.2 form a unified 10-dimensional facial expression parameter vector, referred to as the emotion-conditioned facial descriptor:

***F_emotion_*** = {***θ****_lip_*, ***θ****_head_*, ***θ****_eye_*} (20)

where

(21) $$\begin{array}{ll} \theta_{\mathrm{lip}} & =[{\theta_{\mathrm{lip}}^{\mathrm{aaa}},\theta_{\mathrm{lip}}^{\mathrm{eee}},\theta_{\mathrm{lip}}^{\mathrm{woo}}],\theta }_{\mathrm{head}}\\ & =[{\mathrm{Rotate}}_{\mathrm{pitch}},{\mathrm{Rotate}}_{\mathrm{yaw}},{\mathrm{Rotate}}_{\mathrm{roll}}],\;\mathrm{and}\;\theta_{\mathrm{eye}}\\ & =[\mathrm{Blink},\mathrm{Eyebrow},{\mathrm{Pupil}}_{x},{\mathrm{Pupil}}_{y}] \end{array}$$

**Figure 8:**
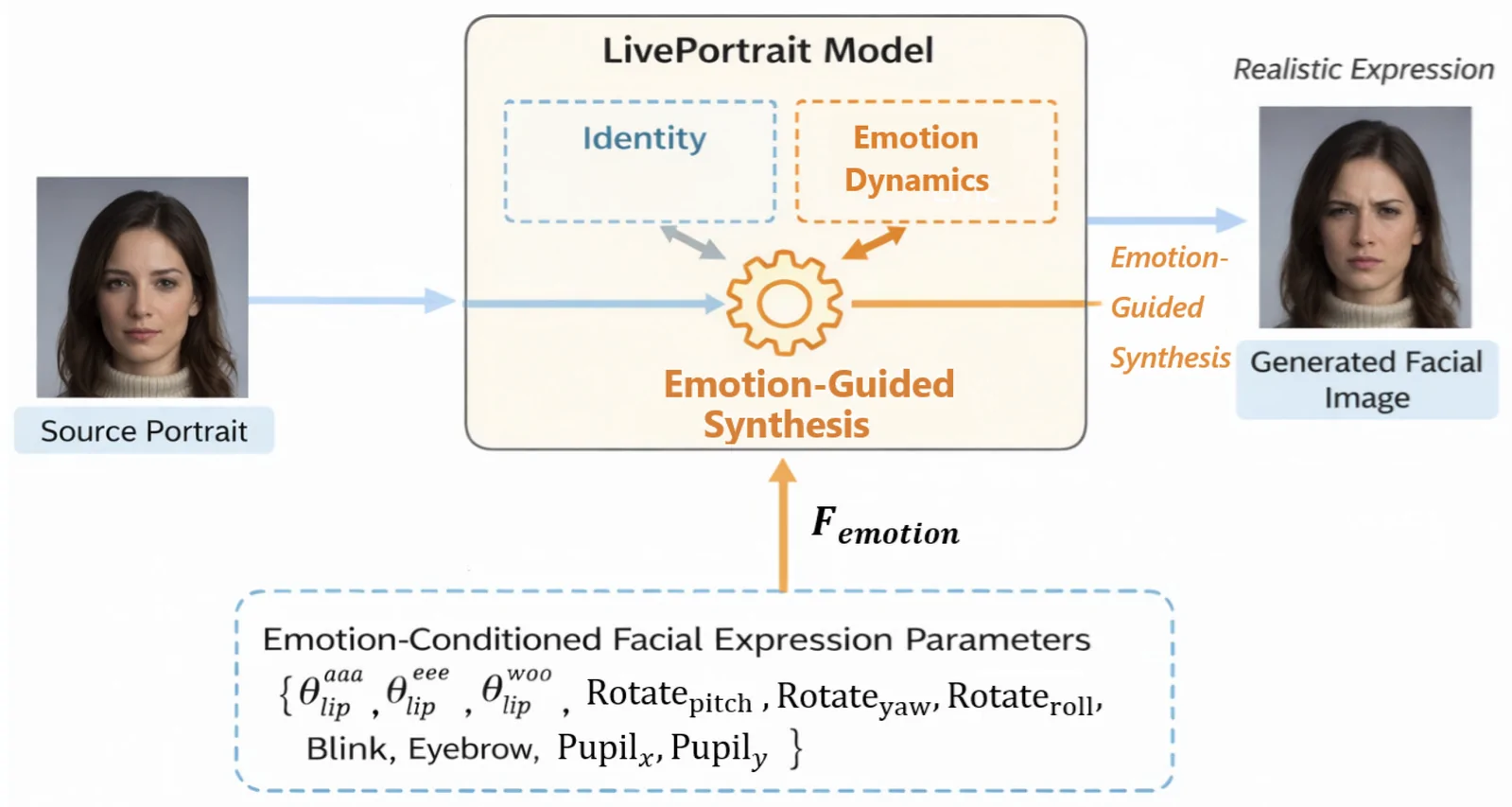
Illustration of the LivePortrait-based portrait synthesis framework used in this study. A source portrait image is provided to the LivePortrait model, while the emotion-conditioned facial expression parameter vector ***F****_emotion_* serves as a motion control signal. The model preserves the identity of the source portrait while modifying facial geometry according to the input parameters—including lip deformation, head orientation, eyelid movement, eyebrow motion, and gaze direction—to generate a realistic portrait image reflecting the emotional state of the input speech.

The head and eye parameter vectors, ***θ***_head_ and ***θ***_eye_, are initialized to unity (i.e., all elements are set to 1) in the neutral state. This descriptor provides a compact and interpretable representation of facial expression that can be directly used as a control signal for emotion-guided portrait synthesis (See Figure 8).

In this work, emotionally expressive facial images are synthesized using LivePortrait [4] through its portrait animation functionality and the Expression Editor (PHM) [25] interface. The proposed 10-dimensional emotion-aware facial descriptor, ***F_emotion_***, is applied through the Expression Editor to control the corresponding facial expression parameters. The Expression Editor provides the interface between the proposed facial descriptor and the LivePortrait-based synthesis process, enabling the generation of expressive facial images while preserving the identity and appearance of the source portrait.

In the proposed framework, the emotion-conditioned facial expression parameters described in Section 3.2 are used as control signals for the portrait synthesis model. These parameters modulate key facial components including mouth deformation, head orientation, eyelid movement, eyebrow motion, and gaze direction—thereby producing a portrait image that reflects the emotional state and articulatory characteristics of the input speech.

The generation of temporally evolving facial parameters used for video synthesis is described in the following section.

### 3.3. Animated Facial Expression Parameters

To generate temporally coherent facial motion from speech, the static emotion-conditioned facial descriptor ***F****_emotion_* defined in Section 3.2.3 must be extended into a time-varying representation. While ***F****_emotion_* captures the instantaneous facial configuration associated with the predicted emotional state, natural facial motion requires smooth temporal evolution that reflects the dynamic characteristics of the speech signal.

To this end, we introduce Animated Facial Expression Parameters (AFP), which are obtained by modulating and interpolating ***F****_emotion_* over time according to the temporal dynamics of the input speech. In particular, variations in speech energy and rhythm are used to control the temporal progression of facial expression parameters, enabling smooth and temporally coherent facial expression transitions synchronized with the input speech signal. The resulting AFP sequence serves as a frame-wise control signal for the portrait synthesis model described in Section 3.2.3, allowing the generation of a sequence of portrait images that evolve consistently with both the articulatory and emotional characteristics of the speech signal. The detailed formulation of the temporal dynamics and the AFP generation process is described in the following subsections.

#### 3.3.1. Speech-Driven Temporal Dynamics

**Figure 9:**
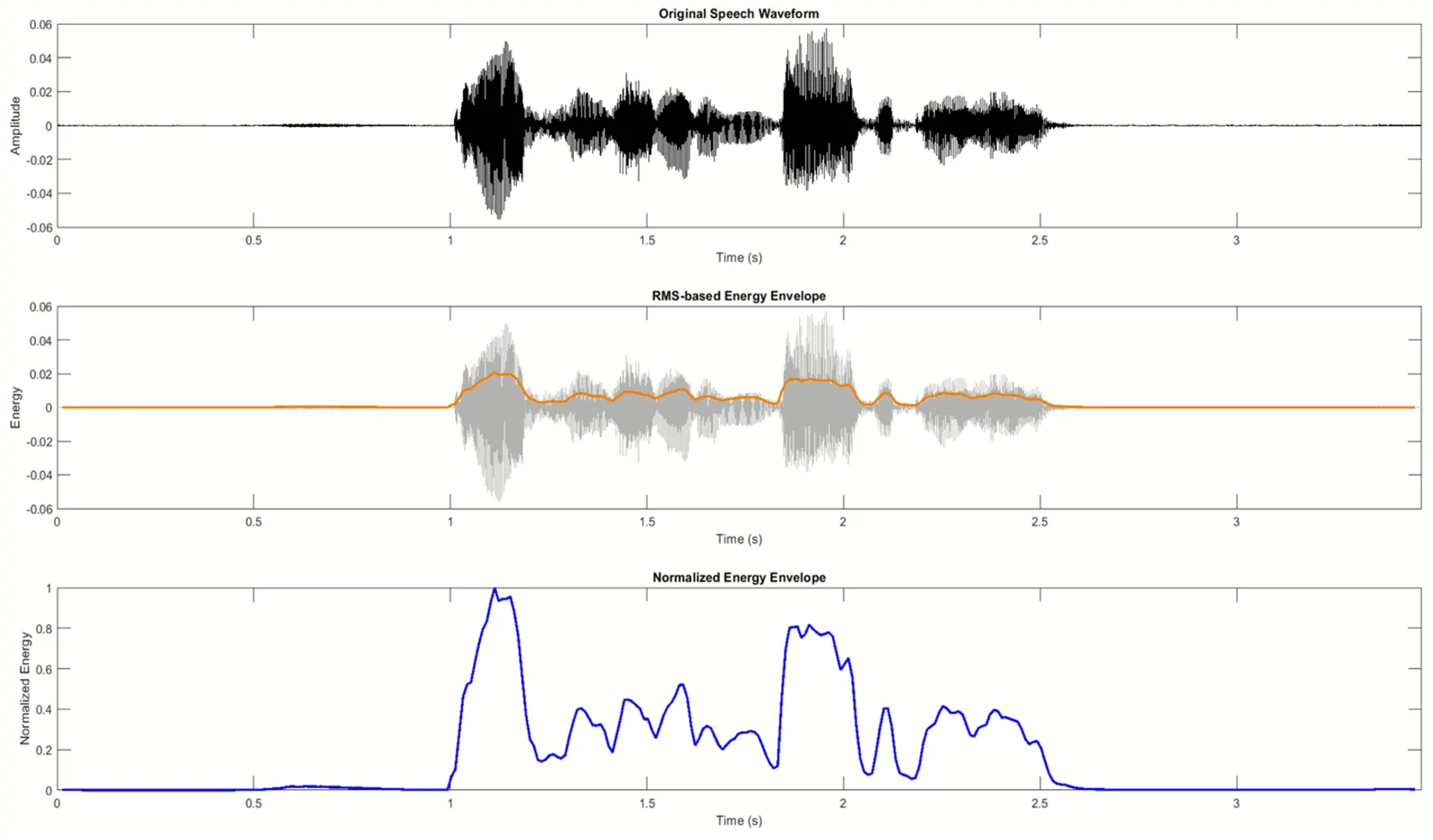
An example of speech-driven envelope extraction for Animated Facial Expression Parameters.

To generate temporally coherent transitions of facial expressions from speech signals, we introduce Animated Facial Expression Parameters (AFP), which modulate facial expression parameters according to the temporal dynamics of speech energy. To capture these temporal dynamics, we employ the envelope of the speech signal, which provides a smooth representation of short-time energy variations. In particular, the temporal modulation is guided by the RMS (root-mean-square) envelope, which effectively reflects the intensity of speech over time. Speech signals inherently exhibit rapid oscillations due to vocal fold vibrations and high-frequency acoustic components. As a result, directly using the raw waveform to drive facial motion parameters can lead to unstable and jittery facial behavior. Therefore, a smoother representation that captures the temporal evolution of speech intensity is required for stable facial control. Such a representation can be obtained through envelope extraction, as emotional dynamics in speech are largely conveyed by variations in energy and prosody. In this study, the RMS-based envelope is adopted as a measure of speech energy due to its robustness and effectiveness in capturing perceptually relevant intensity variations. In addition to providing a smooth representation of short-time energy, RMS energy is less sensitive to signal polarity and high-frequency noise, and has been widely used in speech processing as a reliable indicator of amplitude dynamics [26,27]. Figure 9 illustrates the speech-driven envelope extraction process used in the proposed framework. The top panel shows the original speech waveform, characterized by rapid oscillations. The middle panel depicts the RMS-based energy envelope (orange curve) overlaid on the waveform, capturing the temporal variation in speech intensity. The bottom panel presents the normalized energy envelope, which captures the temporal variation in speech energy while suppressing high-frequency fluctuations. This normalized envelope is used as a control signal for modulating the static facial expression parameters.

The RMS-based envelope *E*_RMS_[*n*] is computed from the input speech signal as:

(22) $$E_{\mathrm{RMS}}\left[n\right]=\sqrt{\frac{1}{N}\sum_{k=0}^{N-1}x_{\mathrm{in}}^{2}\left[n-k\right]}\;n:0\sim N-1$$

where

- *x_in_*[*n*]: amplitude of the input speech waveform at sample index *n*,
- *n*: discrete-time sample index,
- *N*: window length (samples),
- *f_s_* = 48 kHz: sampling frequency.

In this study, the RMS envelope is computed using a sliding Hamming analysis window of 40 ms with a hop size of 20 ms at a sampling frequency of 48 kHz, corresponding to 1920 and 960 samples, respectively.

To further suppress short-term fluctuations, a five-point moving average is applied to the resulting RMS envelope. Each smoothed value is calculated by averaging five neighboring RMS envelope values, providing a smoother representation of temporal speech energy variations for subsequent AFP generation.

The normalized envelope $\hat{E}[n]$ is then defined as:

(23) $$\hat{E}\left[n\right]=\frac{E_{\mathrm{RMS}}\left[n\right]}{\underset{k}{\max}\left(E_{\mathrm{RMS}}\left[k\right]\right)}$$

which satisfies $\hat{E}[n]\in [0,1]$. The normalized envelope reflects the temporal variation in speech intensity and serves as a control signal for modulating facial expression parameters in the subsequent AFP generation process.

#### 3.3.2. AFP Generation via Envelope-Based Interpolation

To generate temporally varying facial expression parameters, the static descriptor ***F_emotion_*** is modulated using the normalized speech envelope $\hat{E}[n]$. The envelope-based interpolation ensures that facial motion intensity follows the temporal energy profile of speech. This process produces the AFP.

(1) Animated Lip Parameters

The animated lip parameter vector is defined as:

(24) $${\hat{A}}_{\mathrm{lip}}\left[n\right]=\theta_{\mathrm{lip}}\;\hat{E}\left[n\right],\;\mathrm{where}\;\theta_{\mathrm{lip}}=\left\{\theta_{\mathrm{lip}}^{\mathrm{aaa}},\theta_{\mathrm{lip}}^{\mathrm{eee}},\theta_{\mathrm{lip}}^{\mathrm{woo}}\right\}$$

(2) Animated Head–Eye Pose Parameters

Since the head and eye parameters must lie within predefined ranges (Table 2), the normalized envelope is used to interpolate between the minimum and maximum allowable values. For the animated head parameters:

(25) $${\hat{A}}_{\mathrm{head}}\left[n\right]=\hat{E}\left[n\right]\cdot \theta_{\mathrm{head}}^{\max}+\left(1-\hat{E}\left[n\right]\right)\cdot \theta_{\mathrm{head}}^{\min}$$

and for the animated eye parameters:

(26) $${\hat{A}}_{\mathrm{eye}}\left[n\right]=\hat{E}\left[n\right]\cdot \theta_{\mathrm{eye}}^{\max}+\left(1-\hat{E}\left[n\right]\right)\cdot \theta_{\mathrm{eye}}^{\min}$$

where ***θ_head_*** = [*Rotate**_pitch_***, *Rotate**_yaw_***, *Rotate**_roll_***], ***θ_eye_*** = [*Blink*, *Eyebrow*, *Pupil**_x_***, *Pupil**_y_***], and ***θ****^min^*, ***θ****^max^* denote the maximum and minimum allowable values of each parameter, as specified in Table 2.

This interpolation ensures stable and physically plausible head and eye motion.

(3) AFP Sequence

The resulting AFP sequence is defined as:

(27) $$\hat{A}\left[n\right]=\left\{{\hat{A}}_{\mathrm{lip}}\left[n\right],{\hat{A}}_{\mathrm{head}}\left[n\right],{\hat{A}}_{\mathrm{eye}}\left[n\right]\right\}$$

**Figure 10:**
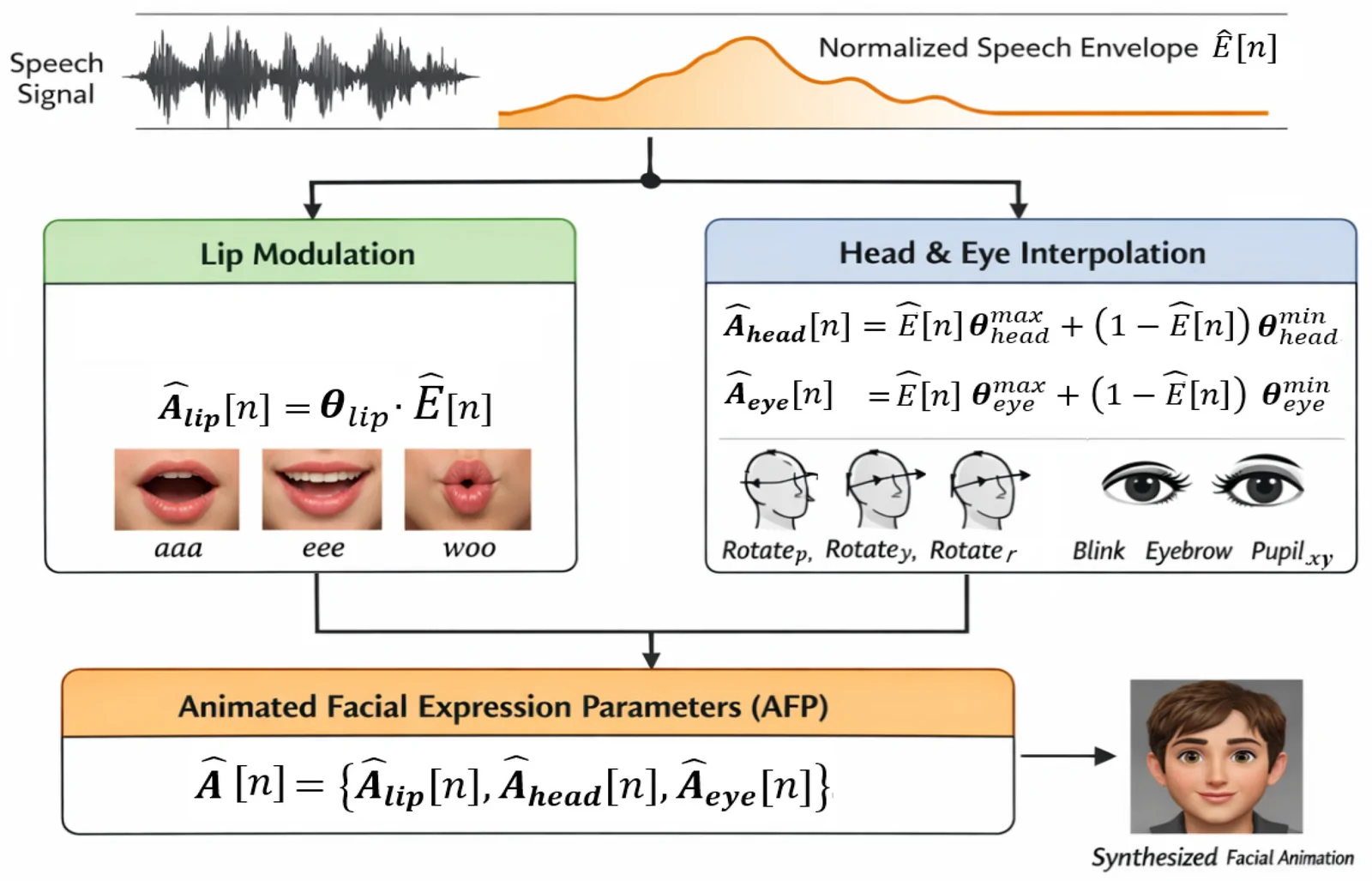
AFP generation via envelope-based interpolation of speech-driven parameters.

The AFP sequence $\hat{A}\left[n\right]\in R^{10}$ serves as a frame-wise control signal for the portrait synthesis model described in Section 3.2.3 by modulating static facial expression parameters according to the temporal dynamics of speech energy, enabling the generation of a sequence of portrait images that evolve consistently with both the articulatory and emotional characteristics of the speech signal (See Figure 10).

## 4. Experimental Results

This section presents a comprehensive evaluation of the proposed speech-driven facial expression synthesis framework. The experiments are designed to evaluate not only the overall emotion classification performance of the SER module but also the contribution of individual acoustic features through systematic ablation studies. Both quantitative and qualitative analyses are provided to demonstrate the effectiveness, robustness, and temporal consistency of the proposed framework.

### 4.1. Experimental Configuration

This subsection describes the experimental configuration used to evaluate the proposed speech-driven facial expression synthesis framework. Since the emotion-conditioned facial descriptor presented in Section 3 relies on accurate emotion estimation, particular emphasis is placed on evaluating the Speech Emotion Recognition (SER) module. The following subsections describe the dataset, audio preprocessing procedure, and acoustic feature extraction process used throughout the experiments.

#### 4.1.1. RAVDESS Dataset

The proposed SER model is evaluated using the Ryerson Audio-Visual Database of Emotional Speech and Song (RAVDESS) [28], a widely used benchmark dataset for emotional speech analysis and affective computing research. The dataset consists of speech recordings performed by professional actors expressing multiple emotional states with a balanced class distribution.

In this study, only the speech modality of the dataset is utilized. Although the original RAVDESS dataset contains additional emotion categories, emotionally ambiguous classes such as calm and disgust are excluded to improve label consistency and semantic correspondence for facial expression synthesis. Consequently, the final emotion set used in this work is defined as *emotion* = {*neutral*, *happy*, *anger*, *sad*, *surprise*, *fear*} [28]. All speech recordings are sampled at 48 kHz and stored in uncompressed WAV format. The dataset is randomly divided into training and testing subsets using a 70:30 split while preserving class balance across all emotion categories.

#### 4.1.2. Audio Preprocessing and Augmentation

To ensure consistent input length, all speech signals are normalized to a fixed duration of 5 s. Utterances shorter than 5 s are symmetrically zero-padded, whereas longer utterances are center-cropped. Furthermore, stereo recordings are converted to mono according to

(28) $$x_{\mathrm{in}}\left[n\right]=\frac{\left(x_{L}\left[n\right]+x_{R}\left[n\right]\right)}{2}$$

where *x_L_*[*n*] and *x_R_*[*n*] denote the left and right audio channels, respectively.

To improve model robustness and generalization, data augmentation is applied to each training data sample. The augmentation strategy includes:

- Time shifting within ±0.5 s;
- Time stretching with a speed factor ranging from 0.8 to 1.2.

Prior to time shifting, zero-padding of 0.5 s is appended to both ends of the speech signal to prevent truncation of speech content during augmentation. These augmentation techniques expose the network to variations in temporal alignment and speaking rate, thereby improving robustness to real-world speech variations.

#### 4.1.3. Acoustic Feature Extraction

Acoustic features were extracted using a high-resolution audio feature extractor configured with a sampling rate of 48 kHz, a Hamming window length of 40 ms, and a hop size of 20 ms. Based on the exhaustive ablation study presented in Section 4.2.1, the following 59-dimensional acoustic feature set was selected for the final SER model configuration:

- 13 MFCC coefficients (spectral envelope information) [20],
- 13 delta-MFCC coefficients (temporal dynamics) [20],
- 1 pitch feature (prosodic information),
- and 32 Mel-spectrogram coefficients (spectral energy distribution) [10].

As described in Section 3.1.1, each speech utterance is represented as a normalized feature (*F*)–time (*T*) matrix:

(29) $$\hat{X}\in R^{F\times T}$$

where *F* = 59 denotes the number of acoustic features and *T* = 249 denotes the number of temporal frames.

The selected feature configuration combines spectral, temporal, and prosodic information, providing a compact yet discriminative representation for emotional speech analysis. The effectiveness of this feature combination is quantitatively validated through the ablation experiments presented in Section 4.2.1.

### 4.2. Quantitative Performance Evaluation

This subsection quantitatively evaluates the performance of the proposed Speech Emotion Recognition (SER) model through feature ablation experiments and confusion matrix analysis. Unlike conventional SER approaches that assign a single emotion label to an entire utterance, the proposed model predicts frame-wise emotion probability distributions at a temporal resolution defined by a 40 ms Hamming analysis window. This frame-level prediction enables continuous modeling of temporal emotion dynamics and provides the emotion-conditioning signals required for the Animated Facial Expression Parameter (AFP) generation process described in Section 3.3.

#### 4.2.1. Feature Ablation Study

To evaluate the contribution of individual acoustic features, an ablation study is conducted using different feature combinations, as summarized in Table 3.

The results indicate that MFCC features alone provide limited discriminative capability for emotion classification. Incorporating delta-MFCC features yields a moderate performance improvement, demonstrating the importance of short-term spectral dynamics in emotional speech analysis. A further and more substantial performance gain is observed when pitch information is included, highlighting the critical role of prosodic cues in distinguishing emotionally expressive speech. The highest classification performance is achieved using the combination of MFCC, delta-MFCC (Δ), pitch, and Mel-spectrogram features. This result suggests that spectral envelope information, temporal dynamics, prosodic variation, and spectral energy distribution provide complementary information for emotion discrimination.

In contrast, the inclusion of additional higher-level spectral descriptors, such as spectral entropy and spectral roll-off, does not consistently improve performance and occasionally leads to performance degradation. This observation indicates that these features may introduce redundant or less discriminative information for emotional speech modeling. Overall, the ablation study demonstrates that a carefully selected set of low-level acoustic descriptors provides a more effective representation for speech emotion recognition than increasingly complex feature configurations.

#### 4.2.2. Emotion Classification Analysis

**Figure 11:**
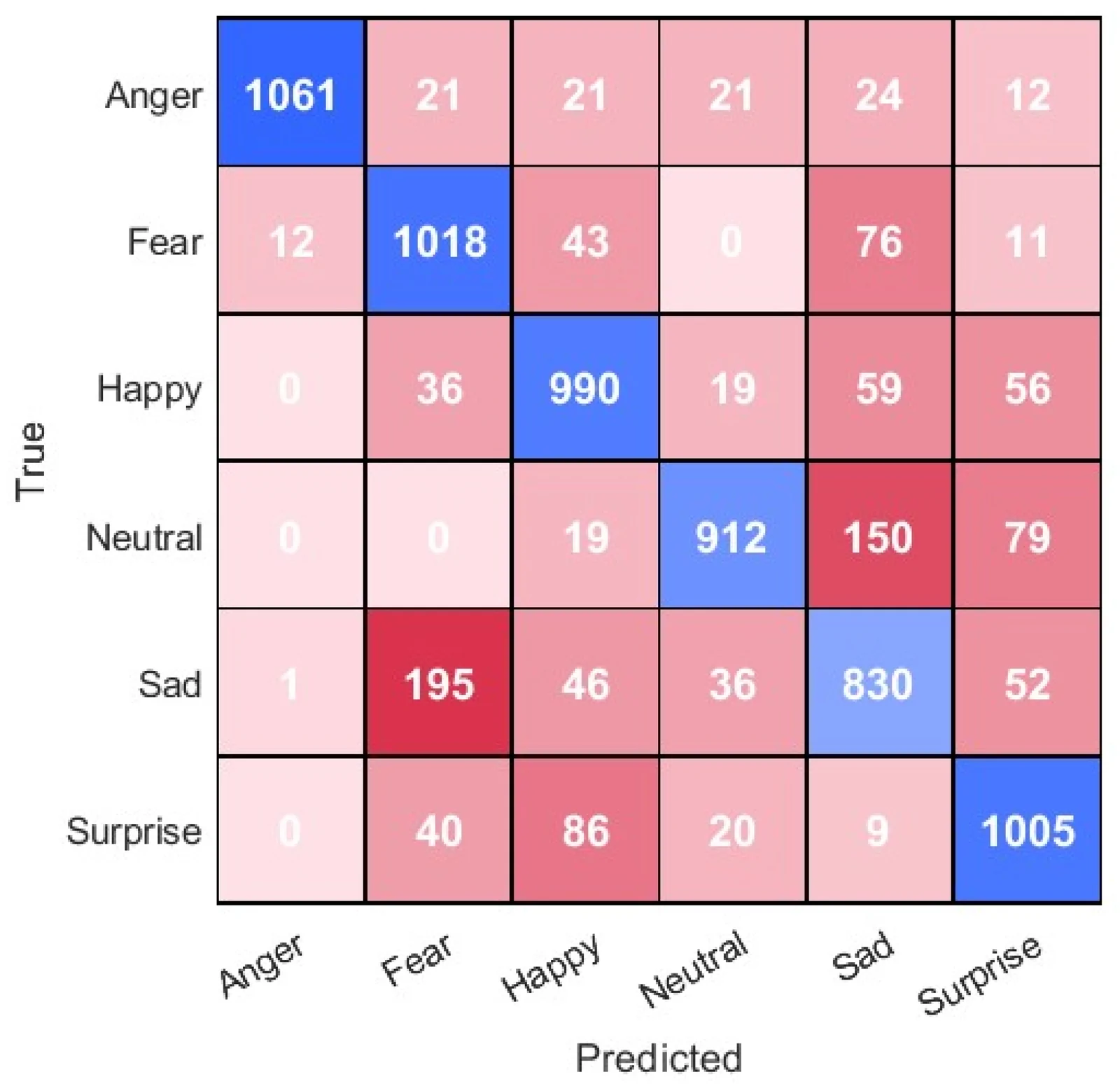
Confusion matrix of the proposed Speech Emotion Recognition (SER) model evaluated on the RAVDESS dataset. The diagonal cells represent correctly classified samples, while the off-diagonal cells represent misclassifications. Color intensity indicates the number of samples in each cell.

Figure 11 presents the confusion matrix of the proposed SER model evaluated on the RAVDESS dataset. Overall, the model exhibits strong diagonal dominance across all emotion categories, indicating that the majority of speech samples are correctly classified.

Among the six emotion categories, anger, surprise and fear achieve relatively high classification accuracy with clear inter-class separability. In particular, anger and surprise exhibit high true-positive rates with minimal confusion with other categories, which can be attributed to their distinctive acoustic characteristics, including elevated energy levels and pronounced pitch variation.

Most misclassifications occur among neutral, sad and fear where acoustic overlap is more prominent. For example, neutral speech is frequently confused with sad and surprise, suggesting that low-arousal speech segments may share similar prosodic characteristics, making discrimination more challenging.

Likewise, sad is relatively frequently misclassified as fear, with 195 samples (approximately 17% of sad samples) incorrectly classified. This confusion suggests that the two emotions may exhibit similar spectral and temporal characteristics in certain utterances. Nevertheless, from the perspective of subsequent AFP generation, similarities between the facial expressions associated with these two emotions may partially mitigate the perceptual impact of such misclassifications.

In addition, happy speech is sometimes misclassified as surprise or fear. This behavior may arise from shared high-pitch and high-energy characteristics, particularly in emotionally expressive speech segments where emotional intensity overlaps across categories.

Despite these confusions, the overall prediction distribution demonstrates that the proposed model effectively learns discriminative emotional representations from speech signals. The strong diagonal structure of the confusion matrix confirms that the network captures meaningful emotional characteristics rather than relying on dataset bias or overfitting to dominant acoustic features.

These results further support the suitability of the proposed SER model as the emotion-conditioning module for the subsequent facial descriptor and AFP generation stages.

### 4.3. Qualitative Evaluation of Speech-Driven Facial Expression Synthesis

**Figure 12:**
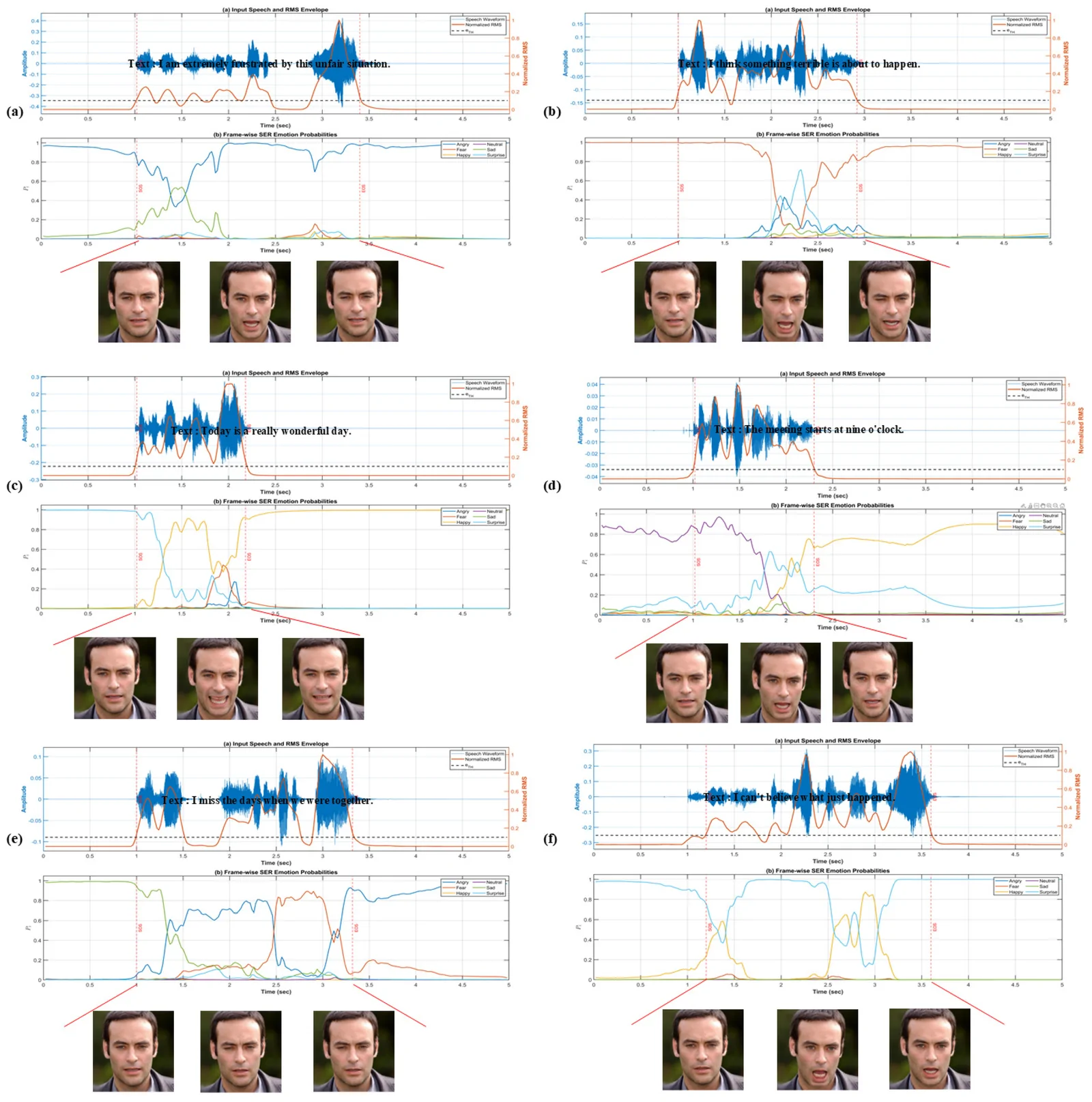
Qualitative results of the proposed framework using a male source image for six representative emotional speech samples: (**a**) Anger.wav, (**b**) Fear.wav, (**c**) Happy.wav, (**d**) Neutral.wav, (**e**) Sad.wav, and (**f**) Surprise.wav.

**Figure 13:**
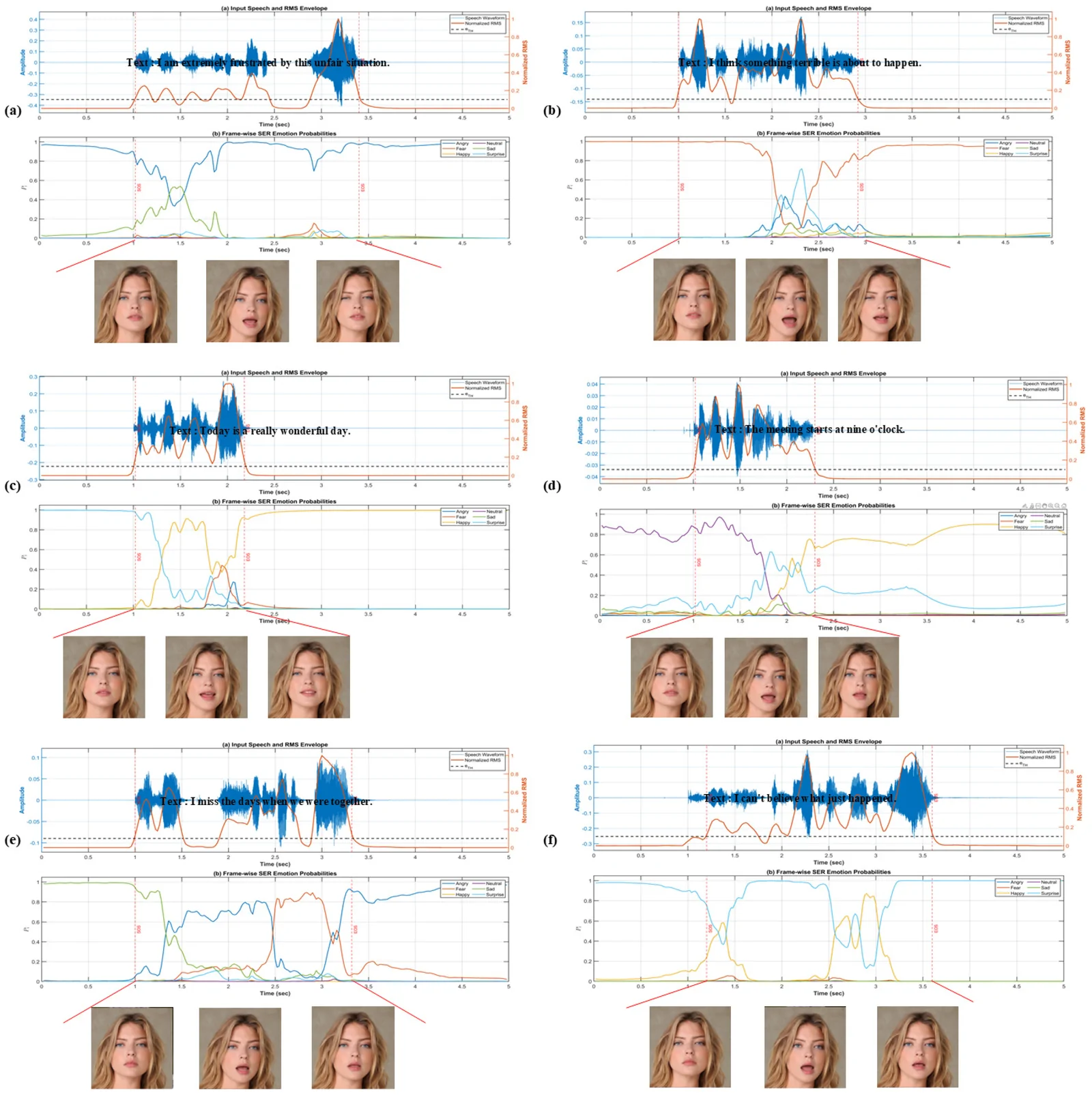
Qualitative results of the proposed framework using a female source image for six representative emotional speech samples: (**a**) Anger.wav, (**b**) Fear.wav, (**c**) Happy.wav, (**d**) Neutral.wav, (**e**) Sad.wav, and (**f**) Surprise.wav.

**Figure 14:**
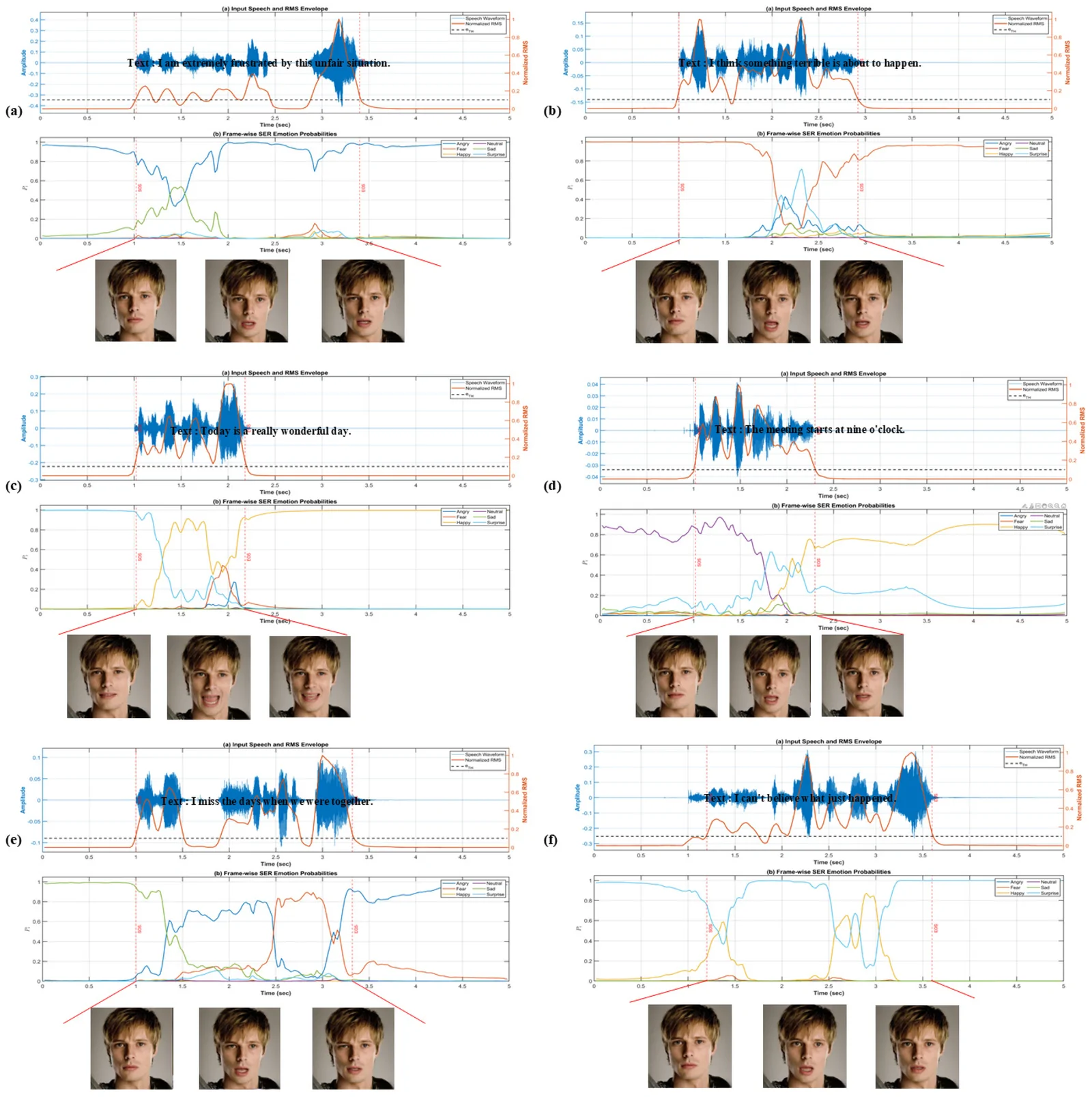
Qualitative results of the proposed framework using a different male source image for six representative emotional speech samples: (**a**) Anger.wav, (**b**) Fear.wav, (**c**) Happy.wav, (**d**) Neutral.wav, (**e**) Sad.wav, and (**f**) Surprise.wav.

To qualitatively evaluate the effectiveness of the proposed framework, we analyze the temporal relationship among speech signals, predicted emotion trajectories, and the corresponding facial expressions generated by the LivePortrait model [4]. Figure 12, Figure 13 and Figure 14 present representative examples with different subjects for six emotion categories, *emotion* = {*neutral*, *happy*, *anger*, *sad*, *surprise*, *fear*}.

For each example, the top panel shows the speech waveform together with its RMS envelope, which captures the temporal variation in speech energy. The middle panel illustrates the frame-wise emotion probability trajectories predicted by the proposed SER model. The bottom panel presents representative facial images synthesized by the LivePortrait model at selected time instances according to the generated Animated Facial Expression Parameters (AFP).

In the experiments, an adaptive threshold is introduced to suppress outlier-confidence emotion probability trajectories as an utterance-level thresholding mechanism, based on the RMS energy of each speech utterance. The threshold is calculated separately for each utterance and remains constant throughout its duration, as illustrated by the horizontal dotted line in Figure 12.

The threshold is defined as

(30) $$e_{\mathrm{th}}=0.1\;\frac{E_{\mathrm{RMS}}\left[n\right]}{\hat{E}\left[n\right]}\;n\in \left[n_{\mathrm{SOS}},n_{\mathrm{EOS}}\right]$$

where *n*_SOS_ and *n*_EOS_ denote the start-of-speech (SOS) and end-of-speech (EOS) sample indices, respectively, and *E*_RMS_[*n*] and $\hat{E}[n]$ represent the RMS envelope and the normalized envelope.

As illustrated in Figure 12a, the predicted emotion probabilities gradually shift toward stronger activation of the **happy** category as the speech progresses. This trend is reflected in the synthesized facial images, where the facial expression becomes increasingly positive and expressive over time. Similar behavior can be observed in Figure 12b–f, corresponding to different emotional speech samples. In these cases, the dominant emotion probabilities evolve consistently with the emotional characteristics of the speech signal, producing facial expressions that exhibit corresponding emotional facial behaviors. More specifically, neutral speech generates relatively stable facial expressions with limited variation, whereas happy and angry speech produce more pronounced facial deformations associated with positive and high-arousal emotional states. In contrast, **sad** speech exhibits comparatively subtle facial variations, reflecting its lower emotional intensity and reduced prosodic dynamics. A clear relationship can also be observed between the RMS envelope and the predicted emotion trajectories. Regions with higher speech energy generally correspond to increased confidence in emotionally expressive categories, indicating that the proposed SER model effectively captures the relationship between acoustic dynamics and emotional intensity. Furthermore, the predicted emotion trajectories evolve smoothly over time rather than exhibiting abrupt fluctuations, resulting in stable AFP generation and temporally coherent facial expression transitions. These observations demonstrate that the proposed emotion-aware framework successfully transfers emotional information from speech signals to facial expressions through the intermediate AFP representation. By integrating speech analysis, frame-wise emotion prediction, envelope-driven temporal modulation, and portrait synthesis, the proposed method produces visually coherent and emotionally responsive facial expressions across a wide range of emotional conditions.

The top panel shows the speech waveform and the corresponding RMS envelope, the middle panel illustrates the frame-wise emotion probability trajectories predicted by the SER model, and the bottom panel presents facial images synthesized from the generated Animated Facial Expression Parameters (AFP).

## 5. Discussion

The experimental results demonstrate that the proposed framework successfully integrates speech emotion recognition, parametric facial descriptors, and envelope-driven temporal modulation to generate emotionally expressive facial motion. By explicitly separating speech-driven articulation from emotion-dependent facial dynamics, the proposed method provides improved interpretability and controllability compared with conventional end-to-end approaches based on implicit representations. One of the key advantages of the proposed framework is the use of Animated Facial Expression Parameters (AFP), which enable smooth temporal transitions by modulating facial parameters according to the speech energy envelope. The qualitative results show that the generated facial expressions evolve consistently with both the emotional content and temporal dynamics of speech, producing visually coherent and emotionally responsive facial behavior. Despite these advantages, several limitations remain. First, the current framework represents emotion using six discrete categories. Although this formulation simplifies emotion conditioning and facilitates interpretable control, human emotions often exhibit continuous and mixed emotional states that cannot be fully represented by discrete classes. Future work may therefore investigate dimensional emotion representations based on Valence–Arousal (V-A) spaces or continuous emotion regression models.

Second, the proposed head–eye descriptor currently uses fixed parameter initialization and envelope-based interpolation. While this approach provides stable facial motion, it does not explicitly model the natural variability of spontaneous head movements, gaze shifts, and blinking behavior observed in real human interactions.

In particular, natural blinks are discrete and transient events that exhibit considerable inter-individual variability in their frequency, duration, and timing. Although the proposed envelope-driven formulation provides a simplified mechanism for controlling eye movements, it does not explicitly characterize the physiological dynamics of spontaneous blinking. Future work will therefore investigate more realistic behavioral models that account for individual differences and the temporal characteristics of blinking, including rapid eyelid closure and reopening.

Incorporating data-driven behavioral models may further improve realism and expressiveness. Third, the proposed framework relies exclusively on speech signals for emotion estimation. In practical human communication, emotional information is conveyed through multiple modalities, including facial appearance, body gestures, and contextual cues. Future work will investigate multimodal emotion recognition by integrating audio, visual, and textual information to improve the robustness of emotion prediction.

Finally, although the proposed framework generates temporally coherent portrait image sequences, its evaluation primarily focuses on qualitative analysis and SER performance. More comprehensive perceptual studies involving human subjective evaluation and quantitative facial animation metrics would provide deeper insights into the quality and naturalness of the generated facial expressions. Overall, the proposed framework demonstrates the effectiveness of combining emotion-aware speech analysis, parametric facial descriptors, and envelope-driven temporal modulation for generating expressive and temporally coherent facial behavior. Future extensions incorporating continuous emotion representations, multimodal perception, and learned facial dynamics are expected to further improve the realism and expressiveness of speech-driven facial expression synthesis.

## 6. Conclusions

This paper presented an emotion-aware speech-driven facial expression synthesis framework based on parametric facial descriptors. The proposed framework integrates three key components: a Speech Emotion Recognition (SER) module for frame-wise emotion estimation, an emotion-conditioned facial descriptor that explicitly separates speech-driven lip articulation from emotion-dependent facial dynamics, and an envelope-driven temporal modulation mechanism that generates Animated Facial Expression Parameters (AFP) for temporally coherent facial motion synthesis.

Experimental results on the RAVDESS dataset demonstrate that the proposed SER model achieves strong emotion classification performance, attaining an accuracy of 83.56% and an F1-score of 83.64%. More importantly, the frame-wise emotion probability representation enables continuous modeling of emotional dynamics, providing effective emotion-conditioning signals for facial expression generation. A key contribution of the proposed framework is the emotion-conditioned facial descriptor composed of a lip descriptor and a head–eye descriptor. The lip descriptor explicitly models speech-synchronous mouth articulation through interpretable vowel-based parameters, enabling controllable representation of mouth opening, lip stretching, and lip rounding associated with both speech production and emotional expression. In contrast, the head–eye descriptor captures complementary emotion-related facial behaviors, including head orientation, eyelid movement, eyebrow displacement, and gaze direction. The integration of these two descriptors enables the generation of facial expressions that simultaneously preserve articulatory accuracy and emotional expressiveness.

Furthermore, the proposed AFP mechanism modulates these facial descriptors according to the temporal energy structure of speech. By combining interpretable facial descriptors with envelope-based temporal modulation, the framework generates facial expressions that evolve smoothly according to both the emotional state and temporal characteristics of the input speech. The resulting AFP sequence serves as an effective control representation for LivePortrait-based portrait synthesis, enabling visually coherent and emotionally expressive facial images while preserving temporal consistency. Taken together, the proposed framework establishes an interpretable bridge between speech analysis and facial synthesis through explicit parametric modeling of speech articulation, emotion-dependent facial dynamics, and temporal speech characteristics. By integrating these components within a unified facial representation, the framework enables controllable and temporally coherent facial expression synthesis that faithfully reflects both the linguistic content and emotional state of the input speech.

## Figures and Tables

**Table 1:** Lip Descriptor.

Parameters		Minimum	Maximum	Description
**vowel**	aaa	−30	120	Vertical mouth opening
	eee	−20	15	Horizontal lip stretching
	woo	−20	15	Lip rounding and forward protrusion

**Table 2:** Head–Eye Descriptor.

Parameters		Minimum	Maximum	Description
**Head**	Rotate_pitch	−20	20	Vertical head rotation (up–down tilt)
	Rotate_yaw	−20	20	Horizontal head rotation (left–right turn)
	Rotate_roll	−20	20	In-plane head rotation
**Eye**	Blink	−20	5	Bilateral eyelid closure intensity
	Eyebrow	−10	15	Eyebrow elevation intensity
	Pupil_x	−15	15	Horizontal gaze displacement
	Pupil_y	−15	15	Vertical gaze displacement

**Table 3:** Ablation study of SER performance using different acoustic feature combinations on the RAVDESS dataset. (MelS: Mel Spectrum, Δ: Delta-MFCC).

Feature	Recall (Sensitivity)						Accuracy	F1-Score
	Angry	Fear	Happy	Neutral	Sad	Surprise		
MFCC	0.8178	0.7031	0.5852	0.6157	0.6378	0.6591	0.6747	0.6731
MFCC + Δ	0.5995	0.4732	0.5260	0.8470	0.6334	0.6287	0.5972	0.6004
MFCC + Δ + Pitch	0.8204	0.7403	0.6442	0.7816	0.6105	0.7505	0.7194	0.7163
MFCC + Pitch	0.9038	0.7028	0.5373	0.9730	0.6142	0.8707	0.7482	0.7435
MFCC + Mel Spectrum	0.8676	0.7818	0.7043	0.6133	0.7420	0.8428	0.7719	0.7649
MFCC + Pitch +Mel**S**	0.8019	0.7491	0.7712	0.8061	0.7240	0.8201	0.7762	0.7740
**MFCC +** Δ **+ Pitch +** Mel**S**	**0.9146**	**0.8775**	**0.8534**	**0.7862**	**0.7155**	**0.8663**	**0.8356**	**0.8364**
MFCC + Pitch + Mel**S** + Entropy	0.8280	0.8096	0.7374	0.8597	0.6826	0.8731	0.7928	0.7894
MFCC + Pitch + Mel**S** + RollOff	0.8359	0.8076	0.7115	0.8155	0.7110	0.7883	0.7749	0.7751

**Note**: **0.** during AFP generation, while maintaining the same SER predictions. Under this configuration, the generated facial expressions remained neutral throughout the animation, regardless of the input speech emotion. In contrast, the proposed RMS-based modulation produced emotion-dependent facial expressions, indicating that temporal envelope modulation is important for generating expressive facial animations.

## Data Availability

The RAVDESS dataset used in this study was obtained from a publicly available Kaggle repository and originates from the original RAVDESS dataset released by Livingstone and Russo. The dataset was accessed and used in accordance with its terms of use. The data generated or analyzed during this study are available from the corresponding author upon reasonable request.

## Abbreviations

The following abbreviations are used in this manuscript:

SER	Speech Emotion Recognition
MFCC	Mel-Frequency Cepstral Coefficients
BiLSTM	Bidirectional Long Short-Term Memory
AFP	Animated Facial Expression Parameters
